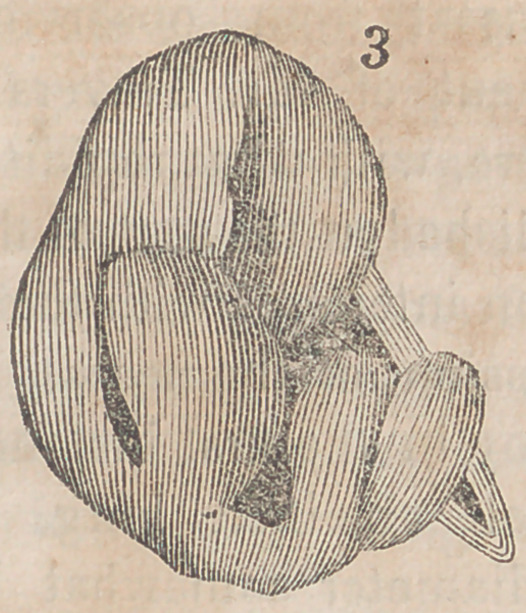# An Experimental and Critical Inquiry into the Nature and Treatment of Wounds of the Intestines

**Published:** 1843-03

**Authors:** Samuel D. Gross

**Affiliations:** Professor of Surgery in the Louisville Medical Institute


### WESTERN JOURNAL

OF

### MEDICINE AND SURGERY.

MARCH, 1843.

### Art. I.—*An Experimental and Critical Inquiry into the Nature and Treatment of Wounds of the Intestines.* By Samuel D. Gross, M. D., Professor of Surgery in the Louisville Medical Institute.

[No. 3.—Continued from page 141.]

### 8.—*Method of Jobert.*

Another mode of treating wounds of the intestines, involving their entire circumference, was proposed in 1822 by Mons. A. J. Jobert, of France, well-known as the author of a very valuable and elaborate treatise on the surgical diseases of the alimentary canal. It is founded on a series of experiments on dogs, and has recently been employed in several instances upon the human subject. The operation is divided into three stages, and the apparatus required for executing it consists of: 1. A pair of probe-pointed scissors; 2. A pair of dissecting forceps; 3. Two double ligatures, carefully waxed, rounded, and from six to eight inches long; 4. Four common sewing needles; 5. Several curved needles for stitching up the outer wound; 6. Sponges, warm water, pledgets of lint, adhesive plaster, square compresses, and a broad bandage.*

The patient, lying on his back near the edge of the bed, is placed in the most favorable manner for the thorough relaxation of the abdominal muscles. The prolapsed bowel is then washed with tepid water, and the edges of the wound, if ragged and bruised, are pared with the scissors. The next step is to dissect off the mesentery for several lines from each end of the injured gut, an operation which is commonly attended with some degree of hemorrhage, which has a tendency, however, to moderate the subsequent inflammation. When very profuse, it may become necessary to secure the divided vessels with temporary ligatures, which are to be removed before the parts are replaced into the abdomen. This constitutes the first stage of the operation.

The second stage consists in the introduction of the needles. To accomplish this the surgeon seizes the upper end with the left hand, while with the right, in which he holds a thread armed at each extremity with a straight and moderate sized needle, he traverses the anterior wall of the intestine from within outwards, at the distance of three lines from the edge of the wound, so as to form a loop with its convexity upwards, and which is now to be intrusted to an assistant. A second thread is then carried in the same manner through the corresponding part of the posterior wall, when the operator, either with his fingers or with a pair of

---

* Traité Theorique et Pratique des Maladies Chirurgicales du Canal Intestinal, par A. J. Jobert; T. i, p. 88. Paris, 1829.

dissecting forceps, inverts the coats of the lower end, and so places the serous surface within the tube. At this moment there is apt to be some contraction of the intus-suscepted parts, which may be allayed, if necessary, by applying to them a weak solution of opium.

Having effected the inversion of the lower end, the surgeon introduces into it the index-finger of the left hand, for the double purpose of preventing it from unfolding itself, and of serving as a guide to the needles. With the thumb and fore-finger of the other hand, he now seizes the two needles of the anterior thread held at the same level, and carrying them along the radial margin of the finger which is in the lower end, he pierces its anterior doubled wall from within outwards, the instruments being brought out at the distance of a line from each other. The needles attached to the posterior thread are conveyed along the ulnar border of the finger, and made to traverse the bowel at a point opposite to the preceding. Then, approximating the injured parts as closely as possible, he withdraws the finger, and gently pulling at the threads, thus gradually introduces the upper end into the lower. The invagination may be facilitated, if necessary, with any smooth, round body. Having restored the bowel into the abdomen, the ligatures are twisted together and placed at the inferior angle of the external wound, which is covered with adhesive strips, a compress and a bandage. On the fourth or fifth day, when the union is said to be sufficiently firm, the threads are withdrawn.

The object of this method is to bring the two serous surfaces of the bowel into contact with each other, and thus promote their re-union. Jobert states that he found in his experiments upon dogs, at the expiration of the twelfth day, a linear cicatrice indicating the place of adhesion between the two ends of the gut, unaccompanied, in the majority of cases, by any particular dilatation of the upper one. Internally there was a sort of artificial valve, the result of the invagination, which floated about in the tube, and formed an inclined plane which allowed a free passage to the alimentary bolus. The mucous membrane appears to have been uninterruptedly continuous. In the five dogs upon which this operation was performed by Jobert, perfect recovery took place. There was no serious disturbance in the functions of the animals, not even in that of defecation.*

There are, I believe, only two cases on record in which this course of proceding was attempted on the human subject. The first is that recently communicated by Mons. Julius Cloquet to the Royal Academy of Medicine of Paris.† The patient was affected with strangulated hernia, attended with mortification of the entire cylinder of the intestine. After having cut away the whole of the sphacelated parts, Cloquet invaginated the divided extremities, and maintained them in apposition by the method of Jobert. As soon as it was ascertained that nothing escaped from the tube on pressure, the bowel was returned, and the abdominal wound secured in the usual manner. When the case was reported fifteen days had elapsed since the operation, without the occurrence of any untoward symptoms, and with the prospect of a speedy cure.

The other case fell under the observation of Professor Berard, who presented an account of it, a few years ago, to the Anatomical Society of Paris. He was called to a female who, in a paroxysm of mania, cut off two feet of her small bowels. He treated the case according to the process of Jobert, but death occurred in thirty six hours without the slightest adhesion between the contiguous surfaces.‡

* Op. cit. p. 91–'2–'3.

† Archives Generales, T. xi, 648.

‡ London Medical Lancet for 1835–'6, p. 45.

The method of Jobert was modified, soon after being made known, by Julius Cloquet, the distinguished anatomist and surgeon. Instead of inverting the lower, and introducing the upper end, he advises simply to pass the needle through the walls of the intestine, a few lines from the division, and to draw the lips of the wound against each other, until the serous surfaces are brought fully into contact. To maintain them in this situation several sutures are required, the ends of which are to be cut off near the knots, when the bowel is returned, and the operation completed.*

Finally, Jobert has proposed the following expedient,† which, it appears, he has also employed with success in his experiments on the inferior animals, though he has not tried it on the human subject. Taking care to distinguish the extremities of the divided gut from each other, he traverses the anterior wall of the upper with a silk thread armed with two needles. Both needles are then carried to the inferior end, and passed separately through the anterior wall from within outwards, when by gentle tractions the operator inserts the extremities into each other, to the extent only, however, of about one line and a half to two lines, without any previous introversion of their edges. The needles are now to be given to an assistant, when, taking another, which should be exceedingly fine, and armed with a very delicate thread, he plaits the serous membrane of the upper end, and afterwards that of the lower. The ligatures are to be tied with a double knot, in such a manner as to invert the inferior extremity, or turn it in upon itself, and thus bring the serous surfaces in apposition with each other. The ends are left hanging out at the external wound. Three sutures made in this way are generally sufficient to preserve the relations of the parts.

Jobert does not consider this method applicable to young subjects, on account of the great fragility of the serous mem-

---

* Jobert, op. cit. T. i. p. 93.—Tavernier's Operative Surgery, translated by the author, p. 277.

† Op. cit. T. i, p. 93.

brane and the facility with which it is torn. Lately, however, he employed it upon a pup with the most perfect success; the animal speedily recovered, and the functions of the digestive canal were executed with their accustomed vigor.

In longitudinal wounds, Jobert employs a procedure very similar to that of Lembert, described in the next section, that is, he inverts the edges, and keeps them in contact by several points of the interrupted suture. The ligatures should be placed so near each other as not to permit any protrusion of the mucous membrane, or, what is the same thing, they should produce, when tied, the most perfect apposition of the serous surfaces. The extremities of the sutures may be twisted together and brought out at the external orifice, as in the method of Le Dran; or, what is preferable, they may be cut off close to the knots, or left hanging out separately. In the former case, they will fall into the cavity of the bowel; in the latter, they may be pulled away at the end of five or six days.*

* Malgaigne, Manuel de Medecine Operatoire, p. 531. Paris, 1837.

I subjoin the following experiments in illustration of Mons. Jobert's first method.

Experiment I.—On the 28th of May, assisted by Dr. Colescott, Dr. Hagan, Mr. Mullen, and Mr. Church, I divided the ileum of a small young dog, and inserted the superior into what was supposed to be the inferior end. The operation was exceedingly difficult and perplexing, nearly half an hour elapsing before it was completed. With all the skill I could command it was impossible to make the ends firmly meet in their entire circumference. No vessels required to be tied. The gut was carefully returned, and the extremities of the two ligatures were brought out at the abdominal wound, which was closed in the usual manner. The animal vomited several times soon after the operation, and refused to take food on the following day, but not water, which he drank with avidity. Late on the third day he died.

The dissection disclosed the following appearances. About two ounces of sero-purulent fluid were contained in the peritoneal sac, which exhibited marks of high inflammation in the greater part of its extent. The omentum covered, and adhered to, the whole of the intestinal convolutions. The small bowels were completely matted together—the lips of the wound were in contact but not invaginated—and the continuity of the tube was established externally by plastic lymph. The ligatures still retained their situation. No fæcal matter was discoverable in the effused fluid, and it was evident that the peritonitis, which destroyed the animal, had been induced by the violence inflicted upon the gut in my efforts to invaginate the divided extremities.

Experiment II.—Immediately after the last experiment I repeated the method of Jobert upon another dog, somewhat larger than the former, and succeeded, after much difficulty, in effecting the invagination. The operation occupied fully thirty-five minutes; it was exceedingly painful, and one of the ligatures lost its hold so much that I was obliged to remove and re-introduce it. Several of the mesenteric arteries bled rather freely, but did not require to be secured. The dog suffered considerably for the first three days, after which he became more lively, and continued so until the fifth of June, when he evinced symptoms of severe indisposition, under which he succumbed on the seventh, the experiment having been performed on the 28th of May. He took food only a few hours before. The ligatures escaped on the fifth day.

The abdominal wound was nearly cicatrized with a small plug of omentum interposed between its lips. The peritoneum exhibited no unnatural redness or vascularity. The small bowel, for about three feet and a half, was enormously distended with gas and fæcal matter, being at least five times as large as in health; its coats were thin, soft, and easily torn; and the mucous membrane was highly inflamed in patches varying in size from a dime to that of a Spanish dollar. The wound was situated near the ileo-cœcal valve with a mass of omentum and the ascending colon intimately adherent to its outer surface. On laying open the tube it was found to be completely obstructed, the inferior end, which was the invaginated one, having become firmly united to the inner surface of the upper, into which it projected in the form of a mammillated protuberance, six lines in length, tapering at its free extremity, and perfectly closed. The part of the small intestine which intervened between the wound and the ileo-cœcal valve was slightly diminished in its caliber, as was also the entire colon: the latter contained scarcely any fæcal matter. In the stomach was a small quantity of undigested food. All the other abdominal viscera were sound.

Such were the results of the above experiments, which are all I have performed with a view of testing this method. It may be supposed that they are not sufficiently numerous to entitle me to deduce from them any general conclusions. I think otherwise. Independently of their unfavorable termination, the difficulty which attended their execution would be enough to deter me, under any circumstances, from resorting to it in the human subject. A practitioner may err through ignorance, but when he does so designedly or despite the most abundant light, neither his own conscience nor the voice of the profession will excuse him. If the method of Jobert were the only expedient of the sort, we might be justifiable in employing it; but, when there are so many others which are all decidedly superior, we should be aware how we give it our sanction. As it is, I do not hesitate to denounce the proposal as unnecessarily harsh in its execution, uncertain in its results, and altogether unwarrantable in the present state of our knowledge. Of the improvement suggested by Cloquet I have no personal knowledge; nor can I say any thing more definite of Jobert's other expedient, described in a preceding page, and which appears to be merely a modification of that of Lembert.

### 9.—*Method of Lembert.*

A very ingenious process of sewing up a wounded intestine, now to be noticed, was proposed in 1825, by Mons. Lembert of Paris, in the second volume of the "Repertoire Generale d' Anatomie et de Physiologie Pathologique." The number of needles to be employed must correspond with the number of sutures designed to be made; they should be long, slender, and armed each with a small but strong and well-waxed thread. The drawings will fully explain the nature of the operation, which is performed in the following manner.* A short stitch, including only the peritoneal and muscular coats, is to be taken up on one side of the wound, dis ant about a quarter of an inch from its edge;

* London Lancet, vol. xi, p. 848.—Johnson's Medico-Chirurgical Review, vol. xxi, p. 299.—Velpeau, Medeciné Operatoire, T. 4, p. 137.—Vidal, Traité de Pathologie Externe et de Medecine Operatoire, T. 4, p. 506.

the needle is then carried across the solution of continuity, and a similar stitch taken up on the opposite side: in this way one suture is to be placed after the other, the interval between each two varying from three to four lines, and when they are all arranged they are to be drawn firmly together and tied with a double knot. By this proceeding, the incision is completely closed,* the serous surfaces are intimately approximated, and the lips of the wound are inverted or turned inwards, forming a kind of valve, about the twelfth of an inch long, within the tube. The ends of the threads being cut off near the knots, the bowel is returned into the abdomen as near as possible to the outer opening, and the case treated as under ordinary circumstances. Mons. Lembert has observed that the sutures usually escape into the cavity of the gut by the seventh or eighth day, after having cut through the parts which they embraced by ulcerative action, and that the plastic exudation which serves as a bond of union between the wounded and the adjacent textures, becomes very quickly organized, and remains a considerable period before it is absorbed.

* The edges of the wound, it will be observed, are only partially united in the drawing, as there are only two sutures, which are not adequate to effect complete apposition and inversion.

Cases are reported in which the proceeding here described is said to have been successfully employed, not only in the inferior animals, but in mortified hernia and wounds of the intestines of the human subject. I subjoin the following, being all I can find upon record.

Case I.—Strangulated congenital hernia of the left side—patient forty-one years of age—bowel accidentally wounded in dividing the stricture—opening an inch and a half long—two sutures—recovery in a month.

This case, related by Jobert, occurred in the hands of Professor J. Cloquet, and is the first of the kind on record.* Nicholas Lejeune, forty-one years of age, of middle height and spare habit, entered the St. Louis hospital of Paris, on the 13th of July 1826, with a strangulated congenital hernia of the left side, for which he had always been obliged to wear a truss. The tumor, which was of large size, was soft and fluctuating, the patient was affected with nausea and occasional vomiting, the pulse was small and frequent, the thirst urgent, the breathing hurried and interrupted, and the abdomen extremely sensitive, with great prostration of strength. Every attempt at the taxis having failed, Cloquet proceeded to perform the operation. The portion of bowel included in the swelling was highly inflamed and enormously distended. The stricture was formed by the neck of the sac. This was divided with the bistoury, when he tried to effect reduction but failed. The instrument was therefore introduced a second time, to enlarge the incision, and as he was

* This case is published by Jobert, (*op. cit. T. i, p.* 280,) as having been treated according to his own method, a circumstance which may be explained by the fact that he claims to be the discoverer of the process above mentioned, and now usually attributed to Lembert. Indeed, the question of priority does not seem to be fully decided; but as this does not impair the merits of the operation, we shall not stop to settle it one way or the other. Mr. Lawrence, (*Treatise on Ruptures, p.* 306,) who quotes the case from Jobert, says it was treated according to Lembert's method; and Velpeau, (*Medecine Operatoire, T.* iv, *p.* 143,) evidently considers it as an example of that kind.

withdrawing it a portion of the intestine, held by one of the assistants, was accidentally opened to the extent of an inch and a half, followed by an escape of gaseous and fæcal matter. With a common needle he immediately sewed up the wound, entering it four lines from the cut edge, and bringing it out at about one line: having carried it in the same manner through the other side, he easily inverted the margins of the aperture, and thus approximated the serous surfaces. Having placed two sutures, he fastened them with a double knot, and, satisfied that nothing escaped, cut off the ends close to the bowel, which he now pushed into the abdominal cavity. Simple dressings were applied to the outer wound, and secured by a T bandage. All the unfavorable symptoms rapidly disappeared, and the man left the hospital cured on the 12th of August.

Case II.—Patient fifty years of age—strangulated crural hernia of the right side—excision of three inches of mortified bowel—number of sutures not stated—death in five or six weeks from the use of indigestible food.

The second case in which this method of enteroraphy was attended with a favorable result, occurred a few years ago in the practice of Professor Dieffenbach, of Berlin.* The patient, a strong, tall husbandman, fifty years of age, had suffered for fifteen days from strangulated crural hernia of the right side. Various attempts had been made at reduction, but without success, by other surgeons, and the probability was that the constricted parts had sloughed, and given rise to

---

* This case was originally published in the "*Wochenschrift für die gesammte Heilkunde,*" Nov. 26, 1836. The British and Foreign Medical Review (vol. iii, p. 517,) in noticing the case, states that the strangulation existed only fourteen days, and that it was inguinal, not crural hernia. Mr. Lawrence (Treatise on Ruptures, p. 362,) however, who obtained his information from the "*Archives generales de Medecine*" for March 1837, makes out the case to have been one of crural hernia, and so does the writer in the London Lancet for June of the same year, and who translated his article from Græfe and Walther's Journal, vol. xxiv, No. 3.

an effusion of fæcal matter. This, indeed, was found, on laying open the swelling, to be the case. The fold of intestine contained in the sac presented, near its upper part, an aperture large enough to admit the thumb. As the fæces did not readily escape, even after the division of the stricture, owing partly to the narrowness of the hernial opening, and partly to the constriction of the gut, the operator destroyed the preternatural adhesions and drew the canal for some distance out of the abdomen. He then excised the whole of the mortified portion, which was at least three inches in length. The corresponding part of the mesentery was removed with a pair of scissors, and a small artery, which was divided in this step of the proceeding, was secured with a ligature, the extremities of which were cut off close to the knot. The open ends of the bowel, which were held by assistants, contracted to such an extent that they would not admit any thing larger than a writing-quill, and the mucous coat was everted. Having united the angular wound of the mesentery with a very fine thread, the lips of the intestinal breach were treated according to the process of Lembert, when the parts were gently replaced within the abdomen. Shortly afterwards castor-oil was administered in large quantities, which was subsequently repeated with croton-oil, and the patient was ordered to remain for sometime on his feet: copious evacuations ensued, with great improvement in all the symptoms. For a few days the treatment was mildly antiphlogistic, and the only remedy given was some castor-oil in laurel-water. The stools soon became natural, the external wound discharged healthy pus, and in a month the patient was so well that he was able to resume his occupation.

The man continued in excellent health for several weeks, when, after severe labor in the field, and the use of very indigestible food, he was suddenly seized with violent pain in the abdomen, vomiting and constipation, under which he died. Two diseased conditions were found within the abdomen. In the left lumbar region a portion of small bowel had coiled around another portion, which it had thus strangulated: above this point the ileum and jejunum were much inflamed, adherent, covered with flakes of lymph, and distended with excrementitious fluid, which was also found in the duodenum and stomach. The gut below the seat of the strangulation was empty and contracted, descending in this state in front of the lumbar vertebræ on the right side, where several convolutions were closely adherent to the walls of the abdomen and to each other. In detaching them, a few drops of pus escaped, and a knot of silk was met with, indicating the exact spot at which the ligature had been inserted, and consequently the place where the tube had been divided. On cutting it open, the parts were found to be united through the medium of a smooth, even cicatrice, half a line broad, and interrupted merely at two points by so many threads, which were still adherent to the surface. There was no perceptible contraction of the caliber of the tube.

Case III.—Accidental wound of the intestine in operating on crural hernia—patient fifty-four years old—two sutures with the ends twisted together and brought out at the external opening—complete recovery.

A third successful example of Lembert's process has been recently published by Mons. Fleury, in a valuable memoir on intestinal sutures in the "Archives Générales de Médecine" for March 1837. In operating on a crural hernia, in a lady fifty-four years of age, a wound was accidentally inflicted on the intestine, which was obscured by adhesions. When the latter had been destroyed, a portion of gut was drawn out of the abdomen, exhibiting a deep mark from the pressure of the crural ring. Mons. Jobert, the operator, determined to close the cut in the intestine by sutures applied in the manner already described, which he accordingly did.* The threads being then united, gentle torsion was made which brought the external edges of the wound together, and placed the serous surfaces in apposition. The bowel was then returned, the ends of the ligatures were left hanging through the outer opening, where they were secured by adhesive plaster, and

* See page 167.

ordinary dressing was applied. The symptoms immediately assumed a more favorable aspect, the bowels acted well on the fourth day, one of the ligatures was withdrawn on the sixth, and the other on the eigth day, and in a month the wound in the abdomen was perfectly cicatrized. At the end of the third month the patient was in excellent health, the functions of the alimentary canal being performed without any irregularity or impediment.*

Case IV.—Gunshot wound of the arch of the colon—three sutures—the ends of the ligatures cut off close to the knots—complete recovery.

A soldier, whose case is mentioned by Mons. Baudens,† was wounded by a ball, which entered three inches to the left of the umbilicus, and passed out at the back not far from the spine. A finger conveyed into the wound readily discovered a large opening in the arch of the colon, which was accordingly drawn out of the abdomen; the edges of the fissure were inverted, and maintained by three points of suture, introduced in accordance with Lembert's method, the ligatures being cut off close to the knots. The man was bled several times at the arm soon after the accident, and subsequently the abdomen was covered with leeches. Under this treatment he rapidly recovered.

Finally, Velpeau alludes‡ to a fifth case in which this operation was attended with favorable results in the human subject. It fell under the observation of Liégard, a French surgeon, but I am not in possession of the particulars.

In the following cases, in which the method of Lembert was employed, death was produced, in two, by causes apparently unconnected with the operation, and in the third by peritoneal inflammation. The two first, which both occur-

---

* British and Foreign Medical Review, vol. iv, p. 512.—Lawrence on Ruptures, p. 307.

† Clinique des Plaies D'Armes et Feu, p. 336. Paris, 1836.

‡ Medecine Operatoire, T. iv, p. 143.

red in the practice of Mons. Jobert, I shall relate as detailed by Mr. Lawrence in his Treatise on Ruptures.

Case I.—Two incised wounds, one transverse, the other longitudinal, the first being united by four, the second by eight points of suture—the ends of the ligatures brought out at the external opening—the patient twenty-three years of age—death in thirty-eight hours from fæcal effusion.

A man, twenty-three years of age, was stabbed in the abdomen with a knife, cutting a portion of intestine, which protruded at the wound, in two places. One of the apertures was transverse, and ten or twelve lines in length; the tunics being completely divided only to the extent of about two-thirds of an inch. It was united by four points of suture, with a common needle and a single thread, carried through the parts in the manner above mentioned. The extremities of the ligatures were then twisted together, which had the effect of approximating the margins of the incision by their external surfaces, and consequently of bringing the opposed serous membranes into contact with each other. These threads were next held by an assistant, while the longitudinal wound, ten or twelve lines in length, was united in a similar manner by eight points of suture. The intestine was then replaced in the abdominal cavity, the ends of the threads being retained on the outside. The patient died in thirty-eight hours in consequence of effusion into the peritoneal cavity from other penetrating wounds of the intestinal tube. The sutures in the wounds of the bowel were covered by a layer of lymph, without any appearance of pus. No thread was visible on the interior; nor was there any interval between the edges of the solutions of continuity. The longitudinal wound formed a projection of two lines in height. The lips of the division still remained in contact even after the removal of the threads; on dragging them apart it was found that they had been united by plastic lymph.

Case II.—Irreducible scrotal hernia—rupture of the bowel by a blow—opening closed by two points of suture—death in less than twenty-four hours.

In this case, the patient, who had been affected with a large irreducible scrotal hernia, received a violent blow on the swelling, followed by symptoms indicating injury of the intestinal canal. On opening the tumor a wound of the bowel was discovered, which was united in the same manner as in the former case, by two points of suture. Death ensued in the night after the operation. The edges of the intestinal wound were found united by plastic lymph, as in the preceding instance.

Case III.—Two gunshot wounds eight inches from each other—excision of the whole of the injured part—ligation of the mesentery—number of sutures not mentioned—death on the third day from fæcal effusion caused by an opening in the cœcum.

Finally, a third unsuccessful case is recorded by Mons. M. L. Baudens, in the work already quoted.* A soldier of the thirteenth regiment of the line was struck by a ball which entered a little to the right of the umbilical region, and passed out behind in the corresponding loin. On introducing the fore-finger into the anterior opening, which was a little larger than usual, the surgeon came in contact with two flattened balls, which had been forced from the man's watch-fob into the abdomen at the moment of the accident. Having extracted these foreign bodies, he conveyed the finger down to the surface of the bowel, which, from its hard and contracted state, he at once supposed to be injured. The affected portion of the tube was then withdrawn, and the simple slit-like aperture which it presented closed with three points of suture. He was about to return the protruded bowel, when, by some exertion of the patient, a fresh portion descended, which was found to have been completely perforated by the ball, eight

---

* Clinique des Plaies D'Armes et Feu, p. 333.

inches from the seat of the other injury. Believing that the best plan would be to remove the whole of the affected part, he accordingly excised it, having previously included the mesentery in a ligature, to prevent hemorrhage. The edges of the new wound were then brought into contact, and retained by Lembert's process; the intestine was reduced, and the ligature just alluded to left hanging out at the external opening. Death occurred on the third day. The sutures were covered with a considerable quantity of plastic lymph, which was already organized; strong adhesions existed between the injured parts and the rest of the small bowel; the perforated omentum had formed extensive attachments; and some coagulated blood was detected between the intestinal convolutions. In prosecuting the dissection, Mons. Baudens discovered an opening in the cœcum with an effusion of fæces, which had been already bounded by the adhesive process; the peritoneum, at this part, was red and very much inflamed, and this at once accounted for the fatal termination of the case.

The late Baron Dupuytren proposed, a few years ago, a modification of Mons. Lembert's method, consisting mainly in the use of the continued instead of the interrupted suture, as recommended by the latter surgeon. The principal advantages attributed to it are, that it is more simple, and that it insures more accurate apposition of the edges of the wound, thereby lessening somewhat the risk of stercoraceous effusion.*

To execute this suture the surgeon takes hold of one end of the bowel with the left thumb and fore-finger, the latter being within the tube, and carries a needle through its tunics a line and a half from the wound, and as near as possible to the mesentery. Leaving a length of thread of about five inches, he intrusts this to an assistant, while he himself grasps the other end, which he treats precisely in the same manner. Having made these two preliminary points, the nee-

---

* Traité Theorique et Pratique des Blessures Par Armes de Guerre, ridigé par Paillard et Marx, T. i, 186. Paris, 1834.

dle is conveyed alternately from one side of the breach to the other, as in the glover's suture, until the entire track is sewed up. The thread being cut off at the same distance from the bowel as at the other angle of the wound, the different stitches are adjusted with a pair of forceps and rendered equally tense throughout. The parts are now returned into the abdomen, and the ends of the ligature brought out at the external incision, where they are to be left for five or six days until the adhesive process is sufficiently advanced, when they may be gently pulled to encourage their separation. If this should be attended with much difficulty, the protruding extremities may be cut off on a level with the skin, and the remainder left to make its way into the interior of the canal. Or, the ends may be cut off in the first instance, and a thread tied to the central loop of the suture before the bowel is restored to the abdomen. By pulling this, when the proper period has arrived, the suture may be easily withdrawn.

The method of Lembert may be further illustrated by the following experiments. They amount altogether to twenty-three in number, and were performed with great care. It will be seen that all, excepting four, had a favorable termination, notwithstanding that the wounds in some of them were of unusual extent. In three, death was produced by peritoneal inflammation, from the escape of fæcal matter; in the other, the animal perished without any obvious or assignable cause. In three of the cases the wound was transverse, in the other longitudinal. In the latter—Experiment II—it was three inches and a half in length, and closed by eleven sutures; death occured on the thirteenth day from the extravasation of fæcal matter, occasioned by the imperfect union of the edges of the incision at its upper angle. All the sutures, except two, had disappeared, the wound was scarcely more than two inches long, and the reparation had been effected mainly through the intervention of an adjacent fold of the small intestine. The caliber of the tube was of the natural size in nearly all the cases in which the parts were examined after death. In a considerable number of them the consolidation of the lips of the wound was remarkably perfect, even at a very early period after the experiment, much more so, indeed, than after the use of the continued or interrupted suture.

### a.—*Transverse Wounds.*

Experiment I.—Complete section of the ileum—four sutures—death in thirty-seven hours from peritoneal inflammation.

June 17, 1842, in the presence of Dr. McDowell, Professor Miller, Dr. Hagan, and Dr. Colescott, I opened the abdomen of a middle-sized and full-grown dog, and exposed a fold of the small bowel, two feet from the ileo-cœcal valve, which was divided entirely across, and the wound closed with four interrupted sutures, equidistant from each other. The animal bore the operation well, but he soon sickened, and died in thirty-seven hours from the time he was removed from the table. The outer wound was feebly united by lymph, and free from omentum. The abdominal cavity contained six ounces of reddish serosity, and the peritoneum, both visceral and parietal, was extensively inflamed. The bowels adhered to the omentum and to each other at various points, and in several of the interstices between them was a small quantity of mucous and fæculent matter. The sutures retained their original situation, and their surface was only partially coated with lymph. On each side of the mesentery the edges of the wound were everted, with a corresponding opening, scarcely two lines in length, through which the alvine fluid had escaped.

Experiment II.—Complete division of the small intestine—six sutures—the animal killed at the end of the ninth day.

Assisted by the gentlemen who witnessed the preceding experiment, I made a transverse section of the small bowel, and retained the divided edges by means of six interrupted sutures, placed at equal distances from each other. The animal, an old slut, bore the operation without much resistance, and suffered apparently very little afterwards. At the end of the ninth day, the cure being considered as established, she was killed. The external wound, which presented nothing unusual, contained a process of omentum: there was no adhesion of the injured part to the adjacent folds of the gut or to the wall of the abdomen, but it was united very firmly to the epiploon, which thus served to point out its situation. The peritoneum was free from inflammation, and the same was the case with the mucous membrane, even in the immediate vicinity of the lesion.

Experiment III.—Complete section of the small bowel—six sutures—fæcal effusion—death from peritoneal inflammation.

The subject of this experiment was a young dog of middle size, in which the bowel, cut entirely across, was sewed up with six sutures, as nearly as possible equidistant from each other. The operation, which was borne well, was performed on the 14th of July, and death occurred on the seventeenth, or about three days and a half after; the animal having all along refused food, and also, during the last forty hours, drink. On examination I discovered about five ounces of thin, dirty colored fluid, evidently of a fæculent nature, with high marks of peritoneal inflammation. Very little adhesion existed between the bowels, except at the seat of the wound, the edges of which were widely separated from each other, all the sutures, save one, having lost their connexion. Small gangrenous patches were seen in different parts of the ileum, and the mucous membrane was deeply inflamed at several points. The external wound was firmly united.

Experiment IV.—Entire division of the small intestine—six sutures—fæcal effusion—death from peritoneal inflammation.

From a small but stout and full-grown dog, I removed a knuckle of the ileum, which was cut completely across, about two feet from the ileo-cœcal valve. The edges were brought together, and maintained in apposition by six sutures, equidistant from each other. The dog struggled a good deal during the operation, from the effects of which, however, he speedily recovered, taking food and drink as usual. At the end of the seventeenth day, being in good plight, and the cure fully established, he was killed. The following appearances were observed on dissection.

The abdominal wound was completely healed, a process of omentum being, as usual, prolonged into it. The injured bowel adhered to a neighboring fold for about three inches, through the medium of a smooth and polished texture resembling serous membrane. A small process of the epiploon was united to the outer surface of the wound, and exhibited a dark modena appearance, from the effusion, probably, of blood at the time of the operation. The omentum was spread over the whole surface of the bowels, which were entirely free from adhesions, except at the place before alluded to. Their movements must therefore have been altogether unimpeded. Internally, the reparation was perfect. The sutures had all disappeared, and the villous edges were not only in apposition with each other, but continuous throughout their entire extent. In fact, the cicatrization could not have been more beautiful or complete. The caliber of the tube at the seat of the lesion was of the natural size.

Experiment V.—Complete division of the upper part of the small bowel—six sutures—the animal killed at the end of the sixteenth day.

The dog employed in this experiment was small but full-grown, and had fasted twenty-four hours. The bowel was divided within fifteen inches of the pyloric extremity of the stomach, and the wound closed by six sutures. No untoward symptoms followed the operation, which was borne without any unusual resistance. He was killed at the end of the sixteenth day, being apparently well but somewhat emaciated. The intestines were every where free from morbid attachments. The omentum adhered around the inner wound, and projected into the outer, precisely as in the preceding experiment. Half of the villous portion of the breach was completely repaired, the remainder only imperfectly, three of the sutures being still retained, and the lips, although in contact, not firmly united with each other. The mucous membrane was of a pale rose color, but not inflamed, and the caliber of the tube natural.

Experiment VI.—Transverse wound six lines in extent—three sutures—the animal killed at the end of a fortnight.

A transverse incision, six lines in length, was made into the lower portion of the ileum, and closed by three points of suture. The dog, a small young tarrier, was scarcely affected by the operation, took food as usual, and was quite playful. At the end of a fortnight he was killed.

A plug of omentum was prolonged into the outer wound, which was nearly cicatrized. The injured bowel adhered to the mesentery and to a neighboring knuckle, by a small quantity of firm, organized lymph, partially transformed into serous texture. Internally, the wound was beautifully repaired, the villous edges being every where in contact, and in the greater part of their extent inseparably connected with each other. One suture, however, still remained, with well-marked traces of the other two. The tube was fully as capacious at the seat of the injury as elsewhere.

Experiment VII.—Transverse section of the small bowel—four sutures—recovery.

In this experiment the small intestine was divided entirely across, and the wound closed by four sutures, which had the effect of completely inverting the serous surfaces, as the tube was unusually narrow. The animal, a small slut, soon recovered from the shock of the operation, and escaped from her box on the seventh day, in good health. She was seen in the street more than a fortnight after; at a period, consequently, when it may be supposed she had entirely recovered.

Experiment VIII.—Complete division of the small bowel—four sutures—death in forty-four hours without any assignable cause.

Having drawn out a fold of the ileum and cut it completely across, I approximated the edges of the wound with the same number of sutures as in the preceding experiment. These had the effect of closing the breach in its entire extent, and of bringing the serous surfaces beautifully together. The dog seemed pretty comfortable for the first six or eight hours, when he began to evince signs of severe suffering, in which he died forty-four hours after he was removed from the table. No attempt at re-union was visible in the outer wound. The edges of the inner wound remained inverted, except at one of its mesenteric angles, where they were slightly separated; scarcely any lymph was discoverable upon them, and the sutures were as distinct and as perfectly in their places as at the moment of their introduction. The peritoneum in the vicinity of the injury was slightly inflamed, but there was no adhesion of the intestines to each other or to the walls of the abdomen. On laying open the tube, the inverted edges were found to form a small valve-like prominence, which was not sufficient, however, to produce any obstruction. What was the cause of death remains therefore a mystery. The probability is that the animal died from the shock of the operation.

Experiment IX.—Complete section of the ileum—six sutures—the animal killed at the end of the twenty-second day.

The subject of this experiment was a large healthy dog that had fasted for twenty-four hours. The small bowel was cut completely across within a foot of the ileo-cœcal valve, and the divided parts were approximated by means of six sutures, equidistant from each other. The dog made some resistance during the operation, and appeared to be considerably exhausted by it. Nevertheless, he rapidly recovered, and was permitted to live till the end of the twenty-second day, when he was killed.

The outer wound was completely healed, and projecting into it was a slender process of omentum. There was no adhesion between the bowels, or between these and the surrounding parts, except at the wound, the surface of which was covered by a mass of epiploon. The tube, which was of the natural size, presented two small sacs or pouches, one above and the other below the seat of the breach, which was perfectly cicatrized, the villous margins being every where continuous with each other. The mucous membrane had a healthy appearance; and the animal, notwithstanding his long confinement and irregular feeding, was in good order.

Experiment X.—Two transverse wounds each half an inch in length—one closed with two, the other with three sutures—the animal killed at the end of the twenty-second day.

From a small dog that had fasted nearly a day, I removed a loop of the small intestine, and made two transverse incisions into it, each six lines long, the first four, the other seven inches from the cœcum. One of these I closed with two, the other with three sutures. The animal bore the operation without flinching, and lived, without any untoward occurrence, until the end of the twenty-second day, when he was killed. The abdominal wound exhibited the usual appearances, that is, it was perfectly cicatrized through the intervention of a plug of omentum. The bowels were free from adhesions, except at the seats of the injury, to each of which was attached a small process of the epiploon. One suture remained in each wound, but it was evident that their presence had not been productive of any mischief, as the continuity of the villous edges had been perfectly re-established. Indeed, the union could not have been more satisfactory. The diameter of the tube was natural.

### b.—*Longitudinal Wounds.*

**Experiment I.—Longitudinal wound two inches in length—seven interrupted sutures—the animal killed on the twenty-fourth day.**

This experiment, together with some of the succeeding ones, was witnessed by Professor Miller and Dr. McDowell. It consisted in making a longitudinal incision, two inches in length, along the convex surface of the small bowel of a middle-sized slut, and in bringing the edges together with seven sutures at equal distances from each other. The animal suffered a good deal for the first twenty-four hours, after which she became comfortable, and so continued until the twenty-fourth day, when, being in good condition, she was killed. The external wound was perfectly cicatrized, and contained no epiploon. The small bowels were matted together, as well as to the omentum, by dense, organized lymph, but they did not adhere to the wall of the abdomen, nor was there any unnatural redness of the peritoneal surface, except at the seat of the injury, where a small ecchymotic spot was visible. On laying open the wounded intestine, the breach was found to be perfectly and beautifully cicatrized in its entire extent, save a small point at each extremity, where the union was not so complete. At one of these places was a small abscess containing a few drops of pus and two ligatures, one partially, the other wholly detached. The injured part adhered firmly to a neighboring fold of the gut, and was in no wise contracted or diminished in its caliber. The adhesion of the villous edges of the wound was more perfect, excepting at the extremities just mentioned, than I ever saw it before in so short a time.

Epperiment II.—Longitudinal wound three inches and a half long—eleven sutures—death on the thirteenth day from fæcal effusion.

This experiment was performed immediately after the last, and with the assistance of the same gentlemen. The wound, extending for three inches and a half along the convex surface of the small bowel, was closed by eleven sutures, as nearly as possible equidistant from each other. The animal was exceedingly fractious, and was much exhausted by the operation, in other respects already sufficiently tedious. For the first few days he was drowsy and listless, refusing such food as was offered him. Before the expiration, however, of the middle of the first week he became more gay, and in a short time appeared to be quite well. He remained thus until the twelfth day, when he was taken sick, and on the thirteenth he expired.

The small bowels were extensively united to each other and to the omentum, a process of which projected into the outer wound. The inner wound had contracted to two inches, and all the sutures, except two, had disappeared. The edges were nearly four lines apart at their centre, elevated, and rounded off, the bottom of the breach, formed by an adjacent fold of the intestine, being covered by a layer of tough, organized lymph. This had given way at the upper extremity of the wound, producing a circular aperture, nearly as large as a five cent piece, through which upwards of eight ounces of thin, fluid, alvine matter had escaped into the peritoneal cavity, where it induced fatal inflammation. The lymph which connected the convolutions of the bowel was firm, dense, and partially transformed into serous texture. The dog was in good condition, and considered out of danger until the occurrence of the accident which carried him off.

Experiment III.—Longitudinal wound one inch and a half long—four sutures—recovery.

From a full-grown tarrier a fold of the small bowel was drawn, and an incision, an inch and a half long, made upon its convex surface, directly opposite the mesentery. The edges of the wound were brought together by four sutures, which had the effect of preventing any protrusion of the villous membrane. The dog suffered apparently no inconvenience from the operation, taking food and drink as before. A month after, the cure being considered as fully established, he was set at liberty.

Experiment IV.—**Longitudinal wound half an inch long—two sutures—the animal killed on the seventeenth day.**

A small pup, not more than about four months old, formed the subject of this experiment. The wound, only six lines long, was made along the convex surface of the intestine, as in the preceding experiment, and closed by two sutures. The animal was a good deal indisposed for the first forty-eight hours, but he gradually recovered his health and appetite, and lived until the seventeenth day, when I had him killed. The external opening was perfectly healed with the intervention of a narrow strip of omentum. The small intestines were slightly adherent to each other, and the internal wound was beautifully cicatrized. Both sutures had disappeared, and the villous portion of the breach was perfectly repaired. No contraction of the injured part was discoverable.

Experiment V.—**Longitudinal wound three-quarters of an inch in length—three sutures—the animal killed at the end of the tenth day.**

The subject of this experiment was a small dog, probably two or three years of age, into the ileum of which, about its middle, I made a longitudinal wound three-fourths of an inch in extent, and brought the edges together by three sutures at equal intervals. The animal bore the operation well, and soon recovered his wonted energy and spirits. He was killed at the end of the tenth day, the cure being considered as established.

The abdominal wound was nearly healed, with a process of epiploon interposed between its inner lips. A small fold of this apron-like membrane was also united to the outer surface of the intestinal wound, and the affected bowel had contracted pretty extensive adhesions to several of the adjacent convolutions. On laying open the tube the villous edges were found to be in close contact with each other, with only a partial re-establishment, however, of their continuity. The sutures still retained their hold, and were buried, as it were, in the substance of the mucous membrane. The latter was perfectly healthy both above and below the seat of the lesion, and the canal itself was in no respect diminished.

Experiment VI.—Longitudinal wound two inches and a half in length—eight sutures—the animal killed at the end of the seventeenth day.

From a very large and healthy dog, shortly after he had eaten a hearty meal, I removed a fold of the upper portion of the jejunum, and made a longitudinal incision, two inches in extent, along its convex surface, directly opposite the mesentery. The edges of the wound were approximated with eight sutures, equidistant from each other. The animal was exceedingly restive during the operation, which was in consequence somewhat protracted, and he lost several ounces of blood. For the first few hours he appeared languid and exhausted, but he rapidly recovered, and was killed at the end of the seventeenth day, being at the time in good condition. The outer wound was perfectly healed with a plug of epiploon between its inner edges. The bowels were free from adhesions, except at the seat of the injury, the surface of which was covered by a small slip of omentum. The caliber of the tube was of the normal size, and the reparation complete. The villous margins of the wound were, however, a good deal more elevated than common; but it was evident that they were every where continuous with each other. The marks of the sutures were still visible. The wound had diminished in length about half an inch.

The mucous coat was perfectly sound, and unpuckered. The arrangement of the parts is tolerably well seen in the drawing. The dark line in the centre represents the ridge formed by the junction of the lips of the wound, which, as has just been stated, were firmly united through their entire extent.

Experiment VII.—Longitudinal wound of the ileum three inches in length—twelve sutures—recovery—the animal killed at the end of the twentieth day.

The subject of this experiment was an old dog, of moderate size, which had fasted for twenty-four hours. The wound was three inches in length, and occupied the lower surface of the small gut, two feet from the ileo-cœcal valve. The sides of the solution of continuity were approximated by means of twelve sutures, placed equidistant from each other. The operation was tedious, and the dog was considerably exhausted before he was removed from the table. During the afternoon he was indisposed to move about, but the next morning the re-action seemed to be completely established, and from this time he rapidly convalesced. He was permitted to live until the expiration of the twentieth day.

On dissection the following appearances were observed. The abdominal wound was entirely cicatrized, and a thick plug of the epiploon intervened between its inner margins. The injured bowel was firmly united to a process of the mesentery, to the omentum, and to the neighboring knuckles, by smooth and organized bands of lymph, strongly resembling the serous tissue. The peritoneal lips of the wound were scarcely discoverable; and as to the villous, they were not only in close contact but inseparably blended together. In fact, the restoration could not have been more perfect. The cicatrice, raised in the form of a narrow ridge, was not more than two inches and a quarter in length, the mucous membrane was no where puckered or diseased, and the tube retained its natural volume. All the sutures had disappeared, though the marks of some of them were still visible, and the villous edges were somewhat elevated, owing to interstitial deposits of plastic lymph. The animal was in good condition, having suffered little or no emaciation from his confinement.

**Experiment VIII.—Two wounds, one longitudinal and the other transverse, the first one inch long, the second three-quarters of an inch—each opening closed with three sutures—recovery—the animal killed at the end of twenty-eight days.**

Into the ileum of a small and very old dog I made two incisions, about eighteen inches from the ileo-cœcal valve. One of the wounds was longitudinal, twelve lines in extent, and situated upon the convex surface of the gut, five inches from the other, which was horizontal, and three lines shorter. Each opening was closed by means of three sutures, equidistant from each other. The dog had fasted for twelve or fifteen hours before the operation, from which he seemed to suffer severely. Notwithstanding this, he rapidly regained his health, and remaining well and in good order, he was killed on the twenty-eigth day.

The outer wound was perfectly healed, without the intervention of the omentum. The bowels had contracted firm and extensive adhesions to each other, as well as to the apron-like lamella just mentioned, but the lymph by which they were produced was quite smooth, organized, and in process of absorption. The sutures had disappeared from both wounds, even to the most minute trace, and the edges of the latter, both serous and villous, were continuous with each other through the whole of their extent and beautifully united. The longitudinal breach was somewhat diminished in length, but the other retained its original size. In both, the cicatrice presented a smooth, rounded, and slightly elevated appearance. The mucous membrane was free from puckers, and the diameter of the tube natural.

EXPERIMENT IX.—Two wounds, each an inch in length—one opening closed with Lembert's, the other with the continued suture—recovery.

In the month of January last, in presence of the medical class, I removed a portion of the small intestine from the abdomen of a small fat dog, eighteen hours after he had taken food, and made two incisions along the convex surface of the tube each fully an inch in length. The lips of one of the wounds were approximated by three points of Lembert's, those of the other by the glover's suture; the contact in each being very close and intimate, so as to prevent the possibility of fæcal effusion. Having cleared away the coagulated blood, the parts were returned into the abdomen, and the edges of the outer wound retained by several points of the interrupted suture. The animal was kept on light diet for the first three or four days, with milk and water for his drink. No untoward symptoms occurring, and the cure being considered as fully established, he was set at liberty on the fifteenth day.

### c.—*Oblique Wounds.*

EXPERIMENT I.—Oblique wound of the small bowel one inch and a half long—five sutures—the animal killed at the end of the twelfth day.

The subject of this experiment, a moderate-sized slut, apparently several years old, had fasted for twenty-four hours. The incision was two feet from the ileo-cœcal valve, and extended obliquely across the gut from one side of the mesentery to within a few lines of the other for one inch and a half. Five sutures, equidistant from each other, were introduced, which had the effect, when tied, of accurately closing the opening in its entire length. No untoward symptoms supervened upon the operation, and the animal was killed at the end of the twelfth day, in good health and condition.

The outer wound was perfectly healed with a portion of omentum prolonged into it. The bowels were entirely free from adhesions, except at the seat of the lesion, which was covered with a small mass of adherent epiploon of a red color. The affected part of the tube was of the natural width, and contained a small quantity of mucous and fæculent fluid. The villous edges were not only in contact with each other but firmly consolidated, their continuity being thoroughly re-established, except at the upper extremity of the breach, where there was a depression about half a line in diameter.

Experiment II.—Oblique wound of the small bowel one inch and three-quarters long—six sutures—the animal killed at the end of the twelfth day.

This experiment was merely a repetition of the preceding. The animal, a small young slut, had fasted for twenty-four hours, and the wound, which was one inch and three-quarters long, extended obliquely from one side of the mesentery to the other. Six sutures were employed at equal intervals. In making the outer opening the bladder was accidentally punctured, followed by a free escape of urine, but no unpleasant symptoms afterwards. At the end of the twelfth day, the animal, being in good health, was killed.

The outer wound had healed through the intervention of a piece of the omentum, as in the preceding experiment. There was no adhesion of the intestines to each other, to the wall of the abdomen, to the other viscera, or to the epiploon, except at the seat of the injury. Two sutures remained in the wound, one being loose, the other slightly attached. The villous edges were separated from each other, without any apparent effort at re-union. The bowel, which retained its natural width, formed a sort of cul-de-sac just above and below the wound, seemingly from the vicious attachment of the omentum. The villous membrane was healthy, and covered with thick, viscid mucus. All the other viscera were sound. The wound in the bladder was beautifully cicatrized.

Experiment III.—Oblique wound one inch long—four sutures—the animal killed at the end of the twenty-second day.

The animal which formed the subject of this experiment was very small and not more than nine or ten months old: he had fasted for twenty-four hours. The wound, one inch long, was situated one foot from the ileo-cœcal valve, and closed with four sutures. Speedy recovery ensued, or, rather the animal did not seem to be affected by the injury, and he was permitted to live till the end of the twenty-second day. The appearances revealed by the examination so nearly resembled those in the last two experiments that it is scarcely necessary to specify them. The outer opening had, as usual, a process of omentum in it, and a small process was also attached to the intestinal wound, which was beautifully cicatrized, the continuity of the villous surfaces being completely re-established. It had diminished about one-fourth in length. The diameter of the tube, however, was natural. The dog was in good order.

Experiment IV.—Oblique wound of the ileum two inches long—six sutures—recovery—the animal killed at the end of the thirteenth day.

The dog was old and of middle size, and made much resistance during the operation, which was consequently somewhat tedious. The experiment was witnessed by Dr. Dodson, Dr. Richard Ferguson, and several other medical friends. The incision, extending obliquely from one side of the mesentery to the other, was two inches in length, and closed by six points of suture equidistant from each other. The dog soon recovered from the effects of the operation, and was allowed to live until the expiration of the thirteenth day.

The outer wound presented nothing unusual. It was pretty firmly cicatrized, with a process of omentum projecting between its inner lips. The injured bowel, intimately connected to several neighboring coils by plastic lymph, was distended with semi-fluid fæcal matter. All the sutures, except two, had escaped; the villous edges of the wound were beautifully united throughout their entire extent, and had an elevated, tumified appearance; there was no puckering of the mucous membrane, and the cicatrice was less distinctly marked than in some of the other cases. The tube retained its natural dimensions. It should have been stated that the wound had diminished in length fully half an inch.

### 10.—*Method of Denans.*

In 1826, Mons. Denans, a surgeon of Marseilles, proposed the employment of three hollow metallic cylinders, in the belief that the serous surfaces of the divided ends of the gut could thereby be kept more effectually in contact than by any other proceeding.* One cylinder is placed into each extremity of the tube, which is then invaginated; the other cylinder, namely, the third, a little narrower than the rest, is next introduced, first into the upper and then into the lower, so as to confine and compress the inverted edges, and serve as a sort of rod for their support. Two of the cylinders are each three lines long, and the other or intermediate one six lines; and each end of the gut is inverted about two lines. To fasten these cylinders Denans employs several points of suture, which embrace the lips of the wound and assist in maintaining them in accurate apposition. When the operation is completed the ends of the threads are cut off close to the peritoneal surface, and the parts returned into the abdomen. The agglutination of the approximated structures is soon effected, and the inverted extremities of the bowel, deprived of their vitality by the pressure of the apparatus, rap-

* Recueil de la Societé de Médecine do Marseille, No. 1. 1826.

idly slough off. The metallic ferules, thus set free, are discharged along with the fæces.

The accompanying engravings will more fully explain the nature of Denans' apparatus and the manner of securing it in the intestinal tube. Figure 1 shows the approximation

1

of the two ends of the bowel, with the small cylinders in their interior; figure 2, the situation of the middle or long

2

ferule; figure 3, a vertical section of the bowel, and the pas-

3

sage of one of the ligatures, to maintain the apposition of the serous surfaces; figure 4, the appearance of the parts after

4

they have been brought together, and the manner of introducing the suture in this stage of the operation.

It is said that this mode of treatment furnished only one successful case in four. In a memoir presented to the Royal Academy of Medicine of Paris, Denans states that in the first experiment the ferules did not pass out of the bowels until seventeen days after the operation. In the second case he wrapped up a small bone in a piece of bread, which was given to the dog, and the instruments were voided at the end of eight days.*

Denans, having recently simplified the above method, now restricts himself exclusively to the three ferules, which are so closely fitted into each other as to obviate the necessity of the suture. The new process is thus described by Dr. Charles Phillips of Liege.† There is, first, a circular row of springs similar to those used as clasps for ladies bracelets. Secondly, the outer ferules are of a conical form, the base of each having a border a line in extent, which, although covered by the reflected intestine, still holds the springs of the inner ring which pass beyond it. By this arrangement the practitioner escapes the difficulty experienced in using the suture. When the first spring is once adjusted, it is only necessary to reflect as much of the bowel as is considered requisite; an advantage which prevents the tumefaction of the edges of the wound and the formation of a fold at the inside of the ferules, which, it is alledged, was the constant cause of the want of success of the original method.

Without having apparently any knowledge of the process of Denans, above described, a very similar practice was proposed, a few years ago, by Mons. Baudens, of France. His account of it is to be found in his work on Gun-shot Wounds, published in 1836. It is certanly less complicated than that of his countryman, but whether it will ultimately be found to possess any decided advantages over it is a circumstance which it is impossible to predict. Baudens uses only one metallic ferule with a ring of gum-elastic, instead of three, as is in the process of Denans. The ferule, moreover, differs from that of Denans in being concave on the back, where it is formed into a groove to adapt it to the gum-elastic ring which embraces it like a clasp. The following is the manner in which the apparatus is applied.

---

* London Lancet for 1834–'5, p. 202.

† Ibid.

The elastic ring is introduced a quarter of an inch within the upper end, the lips of which are immediately inverted, and consequently folded over the instrument, which thus lies in the angle formed by the gut. The ferule is next engaged in the lower end, to the extent of two lines, when the ring is drawn down over it, and the bowel is ready to be reduced into its natural situation. Baudens states that he has employed this method successfully on dogs, and that he would not hesitate, if occasion offered, to resort to it in the human subject.

A distinguished writer in the Dictionnaire de Médecine et de Chirurgie Pratiques, Mons. L. J. Sanson, in summing up the advantages of the different methods of treatment of wounds of the intestinal canal, gives a decided preference to that of Denans. He seems to think that it will insure more perfect apposition of the divided ends, and that it is better calculated also to prevent contraction of the affected bowel, so apt to follow, as he supposes, some of the other procedures. He does not, however, support his arguments by any experiments or observations, and they should therefore be received for what they are worth—merely as so many closet speculations. Mr. Lawrence,* in speaking of this method, very justly remarks that "a patient who could survive the infliction of such surgery must be endowed with great tenacity of life."

### II.—*Method of Reybard.*

The next method that claims our attention is that of Mons. Reybard, of Paris, an account of which was published in 1837, in his "Memoir on Artificial Anus."† The object of it, as set forth by the author, is to effect a temporary obliteration of the wound and to maintain the bowel in strict relation with the wall of the abdomen. For this purpose a ligature, armed with two sewing needles, is passed through a light wooden cylinder, perfectly smooth on its ex-

* Treatise on Ruptures, p. 356.

† See Vidal, Traite de Pathologie Externe, T. 4, p. 503.—Velpeau, Medicine Operatoire, T. 4, p. 135.

terior, and from fifteen to sixteen lines in length by eight or nine in diameter. Thus arranged, and having previously, like Ramdohr, detached a small piece of the mesentery along the concave surface of the tube, the cylinder is introduced into the intestines, where it is fastened by carrying the needles from within outwards through the lips of the wound, about a quarter of an inch from its margin. The extremities of the ligature, crossed and twisted together, are passed, by means of a crooked needle, through the abdominal muscles, at a short distance from the edge of the outer opening. The double thread is now held by an assistant until the surgeon has reduced the bowel; when, taking it in his left hand, he pulls it, and satisfies himself that the injured part is in exact apposition with the abdominal parietes. The operation is completed by separating the ligatures, and tying them over a small compress lying parallel with the inner lip of the wound. In an experiment performed after this method the sutures were cut away at the end of forty-eight hours, and the following morning the wooden cylinder was expelled along with the fæces.*

The nature of this operation will be more fully understood by a reference to the engravings. Figure 1 represents the wooden cylinder.

1

2

Figure 2 is a longitudinal section of the bowel with the cylinder fastened by the ligature.

* Vidal, op. cit. T. 4, p. 503.

Fig. 3 shows the appearance of the parts ready to be returned into the abdominal cavity.

3

Not having repeated the experiment of Reybard, I cannot speak of it from personal observation. It appears to me, however, to be entirely too complicated, to say nothing of the danger which must necessarily arise from the presence of a foreign body, such as he suggests, and which, it may be supposed, might easily be retained in the alimentary canal, causing severe, if not fatal, inflammation, ulcerative absorption, or insurmountable obstruction to the passage of the fæces. It has, moreover, I believe, never been employed in the human subject, and it is obviously nothing but a modification of the process of Duverger, Sabatier, and other surgeons, who recommend the use of a piece of trachea, or other hollow body. Such a proceeding is entirely too mechanical, and would have been better suited to the dark ages than it is to the nineteenth century.

12.—*Method of Amussat, Thomson, Choisy and Beclard.*

As if there were no end to the devices of surgeons for the cure of wounds of the intestines, Professor Amussat, of Paris, has recently proposed another, apparently highly ingenious, which deserves to be mentioned here more on account of its novelty than from any probability that it will ever be employed in the human subject. Like that of Lembert, Denans and Jobert, its object is to place the two serous surfaces in contact with each other, to facilitate the adhesive process, and prevent the effusion of stercoraceous matter. The idea originally suggested itself to Amussat from observing, on repeating the celebrated experiment of Mr. Travers of encircling the bowel with a ligature, with what rapidity the continuity of the tube is re-established at the seat of the constriction, and how little the operation interferes with the comfort of the animal, or the transmission of the fæces. The apparatus which he was led to employ in the first instance was simply a piece of elder-tube, half an inch long, with a narrow central groove, and a diameter somewhat less than that of the intestine. This being introduced into the divided ends of the gut, with the precaution of making the lower overlap the other, as in the operation of Chopart and Desault, a ligature was applied around the parts corresponding with the groove, and drawn with sufficient tightness to cause their strangulation. The result, however, was unsuccessful. The adhesions, from the imperfect approximation of the serous surfaces, failed to acquire the proper degree of solidity, and hence, when the constricted parts were detached, the edges of the wound separated from each other, and the animal promptly perished from the effects of fæcal effusion.

To obviate this accident, Amussat applied to each end of the elder-tube a small conical ferule, which he fastened by means of a small strip of adhesive plaster, the base of the one being turned towards that of the other. By this arrangement he obtained a deep groove, instead of a superficial depression, as in the other contrivance. Two ligatures, six inches long, each passed through a straight needle, and placed opposite each other on the edge at the truncated top of one of the ferules, complete the apparatus by which the strangulation is effected. Thus arranged, the operator introduces the elder-tube into one of the ends of the bowel, where it is secured by passing the needles from within outward through its tunics. The other extremity, held open with several forceps, is then transfixed with both needles together in the same direction, an inch from the lip of the wound, when by means of the two threads the intestine is gradually drawn over the remainder of the foreign body, or, rather, high enough to overlap the other portion to the extent of a few lines. A waxed cord is now applied around the central groove of the apparatus, and drawn with sufficient firmness to strangulate the parts which it embraces. Any redundant substance beyond the cord is to be removed with the scissors, otherwise it will interfere with the union of the serous surfaces, the grand object of the operation. In a few days the constricted parts slough, and the apparatus, being thus set free, is expelled along with the fæces.

Dr. Charles Phillips, to whom I am mainly indebted for this account of the above method, states that it will prove successful in four cases out of five, when performed with proper precaution. I have not deemed it necessary to repeat it on any of the inferior animals, from a conviction that it is obnoxious to the same objections as the process of Denans, without any compensating advantages. Like the operation of Ramdohr, of which, after all, it is merely a modification, it requires a previous separation of the mesentery, to facilitate the invagination of the upper into the lower end; to say nothing of the complicated nature of the apparatus, which cannot always be obtained on the spur of the moment, and which few practitioners will keep on hand in expectation of such an occurrence.

Soon after the above method was made public, Dr. Alexander Thomson, of Paris, suggested certain modifications in the construction of the apparatus, which, however, have only been employed, I believe, on the dead subject. It is impossible, therefore, to say how they might answer in the living. The tube, as improved by Thomson, consists of two pieces, instead of one, which are joined together by an ebony ring, a third of an inch long. The base of each tube is hollow, and marked by a groove two lines in depth by one and a half in width. When united, they present a ridge of two or three lines. "The moveable cone is pierced with two holes at its border for allowing the introduction of two ligatures. Two other waxed threads pass through the substance of the tube, upon which the other cone is fixed. The end of the groove formed by the union of the two cones is made somewhat rough, for the purpose of keeping a more firm hold upon the intestine. The moveable cone is fixed upon a handle, which extends about three quarters of an inch beyond its truncated extremity. At the middle of the handle is a small permanent stud, for the purpose of holding the ligatures which are coiled around it. The extremity of the handle serves to open a free passage into the intestine, until it has reached two-thirds of an inch beyond the base of the cone fixed upon the said handle. Close to the stud are two steel arms, furnished with hooks and springs for securing the intestine. A ligature is then placed over the groove in the base of the cone, and tightened so as to produce strangulation of the intestine, the operator cutting off a portion of the extremity beyond the constricted part. The two ligatures are then loosend, by which the cone is set at liberty, a needle is put on each, and they are passed through the strangulated portion of intestine. The same method having been adopted with respect to the other end of the intestine, the two cones are then united in such a way that the ligatures applied for fixing them may be in immediate contact. They are tied, and cut off near the knots, and the intestine is returned into the abdomen."*

Another modification of Amussat's method was proposed by Mons. Choisy, in a thesis which he presented to the Faculty of Paris, in 1837, for the degree of doctor of medicine. It consists simply in invaginating the divided bowel, and tying it over a piece of trachea. In performing the operation the foreign body is introduced into the superior extremity, where it is fastened by the glover's suture, after which the thread is carried from within outwards across the inferior end, the latter being thus made to cover a portion of the former. The ligature is then applied around the parts, as in Amussat's process, and drawn sufficiently tight to effect their strangulation.† Choisy has performed this operation several times

* London Lancet for 1835, p. 204.

† Velpeau, Medecine Operatoire, T. iv, p. 139.

successfully upon dogs, but whether it has been repeated by other surgeons I have not been able to learn.

Beclard, author of the "Elements of General Anatomy," suggested, many years ago, a mode of treating wounds of the intestinal canal, which, from the success that attended it in some of the inferior animals, he thought might be advantageously applied to the human subject.* It is certainly much more simple than that of Amussat, or the modifications of it by Thomson and Choisy, and if I could be induced to employ any process of the kind, I should unhesitatingly give it the preference. The method under consideration consists in introducing one end within the other, without the intervention of any foreign body, and in encircling them with a ligature drawn with moderate firmness. The serous surfaces are thus brought into close apposition with each other, and the cord, cutting its way through the coats of the intestine, falls in a few days into the tube, where it is discharged along with the fæces.

---

Such is an accurate and impartial account of the various and diversified methods of treatment of wounds of the intestinal canal. Of the estimate to be placed upon them, I have already expressed my opinion, excepting in a few instances, where the facts I have presented are competent to speak for themselves. My conviction is that there are but two sutures which should ever be thought of in the management of this class of injuries, namely, the continued and the interrupted, with the modification of the latter proposed by Lembert. The manner of executing them has been already explained, and it is not necessary, therefore, to say any thing further on the subject in this place.

Whichever of these sutures be employed, the operator should

---

* Chèlius, Traité de Chirurgie, T. i, p. 176. Paris, 1835.

never lose sight of the important principle of closing the opening in the bowel in such a manner as to prevent the escape of fæcal matter. By guarding against this occurrence, the patient will run comparatively little risk of perishing from peritoneal inflammation. When the wound is transverse, and involves the whole cylinder of the tube, I should prefer the continued or common interrupted suture to the method of Lembert, especially in young subjects, in whom the canal is very narrow, or in persons in whom the bowel is over-loaded with fæcal matter at the moment of the injury. In a case of this kind the inverted edges might occasion serious obstruction, from the manner in which they project into the interior of the canal. To longitudinal and oblique wounds, particularly the former, the expedient of Lembert is admirably adapted. The operation is very simple, the sutures easily retain their hold, and the divided edges are more speedily re-united than by any other method.

In reflecting upon the results of the experiments which have been offered in illustration of the use of the above sutures, it should not be forgotten that an operation which is perfectly successful upon an inferior animal, may, when performed upon the human subject, be followed by the worst consequences. In the one, disease is exceedingly rare; in the other, it is not only frequent, but capable of assuming a vast variety of forms, and of sapping the foundations of life when least expected. In the one, peritoneal inflammation is not only uncommon, but, when developed, seldom attains any considerable height; in the other, it is not only easily excited, but extremely apt to terminate fatally. Aware of these facts, the surgeon should always scrupulously guard against the infliction of unnecessary injury; the stitching should be done as gently as possible; and all rough manipulation should be carefully avoided. After the parts have been reduced the external wound should be closed by several points of suture, and every effort made to avert peritoneal inflammation, the great source of danger in injuries of this kind.

It has been alleged that longitudinal do not unite with the same facility as transverse wounds. "There is a curious difference," observes Sir A. Cooper,* "in the facility with which a longitudinal and a transverse wound of the intestine unite. It has been already shown that the transverse heal readily, but with respect to the longitudinal, they have a contrary tendency." In illustration of this assertion, he cites two experiments by Dr. Thomson, of Edinburgh, in which death occurred from the extravasation of fæcal matter, in less than forty-eight hours. The wound in each was an inch and a half long, and closed by four interrupted sutures, with the precaution, in one, of sewing up the intestine with a fine thread. In an experiment performed by himself, in which the incision was of the same length as in the preceding cases, and in which he had recourse to the continued suture, the animal recovered.

My own experience by no means coincides with that of the great English surgeon. We have already seen that, in the twenty-seven experiments above detailed, there were only two deaths, notwithstanding the great extent of the wound in some of them. I have no reason to believe, as Sir A. Cooper apprehends, that the sewing up of a longitudinal wound produces a greater degree of constitutional irritation than that of a transverse one; at all events, I have never witnessed any result of the kind. The experiments which he adduces from Dr. Thomson in support of his opinion were evidently not executed with the requisite precaution. A wound an inch and a half long cannot, as a general principle, be returned with safety into the abdomen with only four interrupted sutures; fæcal effusion would be almost inevitable, especially if the canal happened at the time to be loaded with ingesta, or if the animal were permitted to take much drink or food after the operation. In the second experiment the dog died, not because the parts had not been duly approximated in the first instance, but because the sutures, interrupted as well continued, had lost their hold, and

---

* **Anatomy and Surgical Treatment of Hernia, p. 51.**

thus allowed the wound to gap, and the fæces to escape into the peritoneal sac. In the experiment performed by Sir A. Cooper himself, in which the edges of the solution of continuity were secured by the uninterrupted suture, no effusion could occur, and the consequence was that the animal quickly recovered.

The conclusion, therefore, which I would draw from my researches is, that longitudinal wounds, instead of uniting less easily than transverse, generally adhere with more facility, that they do not produce a greater degree of constitutional irritation, or local disturbance, and that they are not more liable, if as much so, to be followed by contraction of the caliber of the tube at the seat of the injury. The same remarks I consider as applicable to oblique wounds. In nine cases of this kind, treated by the continued and interrupted suture, or by the method of Lembert, there was not a single death, any unusual symptom, or any diminution of the affected cylinder.

### VI.—*Partial and Complete Division of the Intestines.*

In operating for sphacelated hernia it occasionally happens that the constricted bowel contains a small aperture, caused either by the strangulation, or by the efforts which the surgeon is obliged to make to effect the reduction. The gut may also be accidentally wounded by the knife in attempting to divide the stricture, by neglecting to draw down the sac, and holding up the abdominal muscles. A number of examples of this kind are mentioned by authors. One is recorded by Mr. Lawrence in his Treatise on Ruptures, and another, which occurred in the practice of Cloquet, is cited in a previous part of this inquiry. When this accident happens, and the aperture is small, Sir Astley Cooper advises a treatment somewhat different from that which is proper when the tube is mortified in its entire circumference. Instead of excising the affected parts, and bringing the edges together by means of the suture, the surgeon should pinch up the margins of the opening with a pair of forceps, and then include them in a fine silk ligature, drawn sufficiently tight to divide the mucous membrane. The bowel should afterwards be returned to the mouth of the sac, and the case managed upon general principles. The preternatural orifice must not be more than three or four lines in diameter, otherwise it will not only be difficult to prevent the ligature from losing its hold, but the operation will be likely to be followed by undue and injurious contraction of the gut.

The following experiments and cases will exhibit this operation in a more forcible point of view. Of the latter, two occurred in the hands of Mr. Lawrence, the other in those of Sir Astley Cooper, with whom I believe the practice originated, and to whom surgery is indebted for some of its most ingenious and substantial improvements.

Experiment I.—Having opened the abdomen of a small slut, and exposed a fold of the ileum, I made an incision, half an inch in length, along its convex surface, and secured it by means of a strong silk ligature tied firmly round its sides. Some difficulty was experienced in preventing the thread from slipping; it was drawn with considerable firmness, and when the ends were cut off it was found to be nearly concealed from view by the apposition of the serous surfaces. The bowel was then returned, and the outer wound closed in the usual manner. The animal did not appear to mind the operation, which was soon over, and she was permitted to live until the ninth day. It is unnecessary to mention all the particulars of the post-mortem examination. Suffice it to say that the small intestines were slightly agglutinated to each other and to the omentum, and that the latter projected into and assisted in closing the outer wound. The bowel at the seat of the injury was remarkably firm, and presented numerous red points. The ligature had disappeared, and the edges of the wound were about three lines apart at their centre, without any contraction of the caliber of the tube. The bottom of the wound was consequently formed by a neighboring convolution protected only by a thin layer of lymph of a yellow-greenish appearance, from the admixture evidently of bilious matter.

Experiment II.—The incision in this experiment was transverse instead of longitudinal, but of the same extent as in the preceding. It was situated in the small bowel, about two feet from the ileo-cœcal valve, and the difficulty experienced in encircling it was still greater than in the former case. One end of the ligature being cut off near the peritoneal surface, the other was brought out at the external wound, which was closed in the usual way. The animal, a small pup, soon recovered from the shock of the operation, and was killed twenty-three days after, the ligature having been detached towards the end of the first week. The outer wound was completely cicatrized, with a process of omentum adherent round its margins, as well as to the convolutions of the small intestines. The latter were strongly united to each other at several points, particularly at the seat of the injury, which was almost perfectly repaired, the mucous membrane being deficient over a space not exceeding the diameter of a split pea. The bowel retained its normal dimensions, and the animal was in good condition at the time he was killed.

Case I.*—John Shall, sixty years of age, was admitted into St. Bartholomew's Hospital, on the 2d of November, 1826, with strangulated inguinal hernia. The tumor was hard and painful, the abdomen was tender on pressure, and there was a sense of tightness across the navel, with constant nausea and occasional vomiting. The pulse was small and frequent, and the symptoms in all respects urgent. All attempts to replace the parts by the taxis having failed, Mr. Lawrence proceeded to operate eight hours after the bowel had come down. The swelling contained a portion of small intestine in front with a large mass of omentum behind, and the stricture was caused by the neck of the sac, which encircled the protruded tube like a tight cord. On withdrawing

* Lawrence's Treatise on Ruptures, p. 301–3.

the intestine gently, an opening was discovered in it just above the part that had been compressed, and which had probably been made by the bistoury in dividing the stricture. The sides of this aperture, which was very small, being held with the dissecting forceps, a ligature was firmly tied around it, after which the ends were cut close to the knot. A piece of omentum, which had been long protruded, and which it was found difficult to return into the abdomen, was removed with the knife, and the divided vessels, six or eight in number, secured in the usual manner. The integuments were brought together by three or four sutures, assisted by strips of adhesive plaster. Soon after the operation the bowels were evacuated with senna, and blood was twice taken from the arm. On the 6th of November the sutures were removed from the outer wound, and on the 13th the ligatures came away from the omentum. It is needless to add that the patient rapidly recovered.

Case II.—In another case, in which the bowel was wounded, Mr. Lawrence * pursued the same method. It was a large enterocele with the intestines greatly distended and the abdomen so very tense that it was difficult to replace the parts and prevent them from re-descending. The symptoms were not relieved by the operation, and death ensued within two days. The ligature was completely covered by a thin smooth layer of lymph, and so concealed that there was difficulty in finding it: the small wound in the bowel was closed.

Case III.—Joseph Curtis, a butcher, twenty-one years of age, was brought into Guy's Hospital, on the 9th of December, 1808. He had a tumor in his left groin, which was very hard and tense, and gave considerable uneasiness on pressure. Along with this was violent pain in the stomach with vomiting of green bilious matter. Various attempts were made at reduction, but they all failed, and the opera-

---

* Op. cit.

tion was therefore at once determined upon by Mr., afterwards Sir Astley Cooper. About four inches of the small intestines were found in the sac, of a dark reddish color, with the testicle at the lower part. The stricture, situated at the mouth of the sac, was divided in the usual manner; a fluid of a yellowish appearance escaped, and on turning up the gut an opening was discovered, which was immediately laid hold of with a pair of forceps, and tied with a ligature. The parts were then returned, and the abdominal wound secured by five stitches assisted by adhesive strips. The patient bore the operation well, and seemed much better after it. For the first ten or twelve days, however, his sufferings were severe, but he gradually surmounted them, and was discharged cured on the 17th of January, 1809, a little more than three months after his admission.*

In commenting on this case, Sir Astley Cooper uses the following language: "We had the pleasure and satisfaction to see the patient completely recovered from an operation, the circumstances attending which were remarkable, and such as will tend to throw much light upon a subject hitherto but little understood."

The above plan, so happily employed by Sir Astley Cooper and Mr. Lawrence, has doubtless been adopted, if not actually executed, by numerous other surgeons. "Many years ago," says Prof. Gibson,† in speaking of Sir Astley Cooper's procedure, "I performed a similar operation in a case of hernia, and with equal success." Mr. Syme, of Edinburgh, recommends the same practice,‡ which may now, indeed, be considered as being fully sanctioned both by observations on the human subject and experiments on the lower animals.

Such is the treatment which should undoubtedly be pursued by the surgeon when he meets with an aperture of small

---

* The Anatomy and Surgical Treatment of Abdominal Hernia, Part i, p. 45. Second edition.

† Institutes of Surgery, vol. i, p. 119. Philadelphia, 1838.

‡ Principles of Surgery, p. 262. Second edition.

size in the strangulated bowel. When the gangrene, however, involves the entire cylinder of the tube, a different mode of management must be resorted to. Under these circumstances, the affected parts should either be excised, and the edges approximated by the suture; or they should be freely opened, and maintained in contact with the abdominal wound, to afford a ready outlet to the fæces. The experience of the profession has not yet fully determined, I think, which of these methods should be adopted to the exclusion of the other, or whether both are not occasionally justifiable. Several examples have already been cited in which excision was practised with the most complete success. The memorable case of Ramdohr is of this kind. An analogous one is recorded by Baudens, and mentioned under the head of Lembert's process of sewing up wounds of the intestines. The case which occurred in the hands of Dieffenbach is also in point. The sphacelated part was at least three inches in length; the whole of which was removed with the knife, and the divided extremities secured by suture. The man lived nearly a month after the operation, and would have completely recovered but for some imprudence in his diet. In another case four inches of mortified intestine were removed, and the patient, a young man, recovered.* Many examples of a similar description are on record, but it is not necessary to refer to them more particularly in this place.

The practice of excision derives support from what is occasionally witnessed in intus-susception of the intestines, in which large pieces of the tube are detached without any detriment to life. In my museum of morbid anatomy is a preparation of this kind, presented to me by my friend Dr. Dawson, of Ohio, in which a portion of the colon, twenty-nine inches long, was discharged by a child six years of age, who, notwithstanding, made a most rapid recovery. This patient, as I have been recently informed, is still living and

---

* Sir A. Cooper on Hernia; p. 37.

in perfect health, three years after the above occurrence. Thirty-five cases of a similar nature, collected from the writings of different pathologists, have been reported by Dr. Thompson of Europe.* The length of the eliminated pieces varied from six inches to upwards of three feet: they generally involved the whole cylinder of the bowel, and nearly all had a portion of mesentery attached to them. In one instance there was a mesenteric ganglion, in another a process of omentum. The average duration of the disease was between four and five weeks. In twenty-two of the cases the evacuated portion appertained to the small bowel, in the other to the large, or jointly to this and to the former. The cœcum was affected alone in one instance, the colon in two, the jejunum in three, the ileum in eleven.

The following case may be adduced as throwing additional light upon this interesting and important subject. It occurred in the practice of Dr. McKeever, of Dublin, and will be found recorded in the fourth volume of the London Medico-Chirurgical Review.

A young robust woman, after having been in labor for upwards of thirty hours, was delivered on the 29th day of July, with the crotchet, previously to which a rent had taken place high up in the posterior part of the vagina, which extended round the neck of bladder, and communicated freely with that viscus. On the following day, in the afternoon, one of the attendants observed a shining substance hanging from the external parts, which was found, on the fifth of August, when Dr. McKeever first visited her, to be nearly a yard and a half of her small bowel coiled up under her, black, apparently putrid, and full of openings. Her belly at this time was much swollen, and excessively painful; her stomach rejected even the mildest articles of diet; the bowels were still obstinately confined; the pulse was small, intermitting and tremulous; and her countenance

*Edinburgh Medical and Surgical Journal, Oct. 1835.—See also the author's Elements of Pathological Anatomy, vol. ii., p. 260.

was pallid and ghastly: in short, she had every appearance of being in a moribund state. It being too late to return the parts, the treatment was merely palliative. On the following day, the protruded portion of the intestine had a soft doughy feel, was more shrivelled, and, instead of being black and livid, it was of a dirty ash-color. The constitutional phenomena were as before. On the seventh day the mortified parts, measuring precisely three feet and eleven inches, were detached, and the woman was nearly free from alarming and distressing symptoms. The vomiting and hiccough had ceased, her pulse was regular and of good strength, the countenance much improved, and the abdomen, though still much swelled, less tender to the touch. She had also a copious discharge of fæces by the vagina, being the first alvine evacuation she had since her delivery.

From this time she gradually mended. Her countenance improved, the secretion of milk became abundant, and the excrementitious matter was of a healthy color, smell and consistence. Three years after the occurrence of the accident, she could walk a dozen miles without inconvenience, and had become fat. For two years after her confinement she had no discharge whatever from the rectum, the residue of her food being altogether voided by the vagina. About the end of that period, however, she was attacked with violent bearing-down pains, accompanied by tenesmus, and after half an hour's severe suffering, she passed by the natural route a large quantity of dark, pitch-colored fæces, of the consistence of balls of firm wax. It is unnecessary to give further particulars. Suffice it to say that the woman was afterwards safely delivered of a small child, and that the fæces have ever since been discharged in the natural way.

The above case requires no comment. It is in all respects one of the most extraordinary on record, and affords convincing proof that injuries attended with the loss of large portions of the alimentary canal, are not necessarily fatal. Coxe's Museum contains a case, from the London Philosophical Transactions, of a boy who had his bowels protruded, and fifty-seven inches cut off by a cart, who, nevertheless, recovered his health in six or seven months.

To these observations I add the following experiments as having a direct bearing upon the subject under consideration.

Experiment I.—From a small but full grown dog two inches and a half of the ileum were removed, near its junction with the large bowel, after which the edges of the wound were brought together with six interrupted sutures, introduced equidistant from each other, and made with a common needle and fine silk. The extremities of the ligatures were cut off close to the knots, and the parts being restored to their natural situation, the abdominal wound was secured by several stitches. Several ounces of blood—perhaps four or five—were lost during the operation, and the animal appeared to be somewhat faint. In the evening he was dull and drowsy, and indisposed to move about; but in the morning he was observed to be better, and from that time he rapidly recovered. Four months afterwards, being in good health, and the outer wound perfectly healed, he was killed. Externally the bowel was smooth and natural, with no trace whatever of the former injury, excepting the attachment of a very small process of the epiploon. Had it not been for this circumstance it would have been exceedingly difficult, if not impossible, to find the seat of the wound. The mucous membrane was of the natural color; there was not the least contraction of the tube; and the situation of the breach was indicated merely by a very narrow oblique line or depression. No adhesions existed between the bowels or between them and the walls of the abdomen. See pl. fig. 8.

Experiment II.—In a second experiment five inches of the ileum were excised, and the lips of the breach maintained in contact by seven interrupted sutures, with the ends cut off close to the serous surface. The divided mesenteric vessels bled so freely during the operation that it became necessary to secure them with a ligature, which, however, lost its hold in attempting to replace the bowel. The dog, which was small, and not more than about a year old, died in thirty hours from the protrusion of eighteen inches of the small bowel, which was lacerated near its middle, of a dark livid complexion, and apparently sphacelated. Externally the wounded surface was slightly coated with plastic lymph, as well as partially covered with adherent omentum, and the parts above and below were of a deep rose tint. The mucous lining immediately around the seat of the injury was of a purple color; and there was a small coagulum where theligature had slipped from the mesenteric vessels. No fæcal matter had found its way into the peritoneal cavity; the sutures had retained their situation; the lips of the wound were in contact with each other, both internally and externally; and it was obvious enough that the animal had perished from the protrusion and consequent inflammation of the ileum. The cause of this accident was the premature detachment of the stitches in the outer opening.

Experiment III.—Finally, in a third experiment the portion of ileum cut away measured eleven inches and a half. The edges of the divided extremities were brought together, and maintained in apposition by means of the continued suture, made with fine sewing silk, well waxed, and armed with a delicate needle. Several of the mesenteric arteries were surrounded with a ligature, which was brought out at the orifice in the wall of the abdomen. The dog, large, and several years old, became sick soon after the operation, which was both tedious and painful; at the expiration, however, of twenty-four hours he took food, appearing lively and even cheerful. He continued thus until the eighth day, when he was observed to be seriously indisposed, and early on the following morning he died.

On inspection, the inner lips of the wound were found to be in a soft, pouting condition, slightly covered with mucous, but no fæcal matter, and without any perceptible attempt at restoration; the suture was still in its place. Three folds of the intestines were glued together at the seat of the injury, and the parts there were somewhat red, as the effect of inflammation. Numerous petechial spots were observed upon the parietal portion of the peritoneum; and the serous and muscular tunics, both of the small and large bowel, presented, in several situations, a singularly lacerated aspect. The villous membrane in the vicinity of the wound was softened, and covered with a considerable quantity of thick, ropy mucus. The stomach and other organs were healthy. There was no obstruction from fæcal matter, or any contraction of the caliber of the tube.

It will be seen from the foregoing statements that only one of these experiments terminated favorably, namely, the first, in which the excised portion of intestine amounted only to two inches and a half. In the second, the animal might possibly have recovered had not the sutures of the external wound given way, and thus permitted the escape of the bowel, which was subsequently lacerated, and seized with violent inflammation. In the third experiment, in which nearly one foot of the intestine was removed, the dog seemed to suffer severely from the shock of the operation; and, although re-action soon took place, he finally perished, on the ninth day, from the effects of his wounds. How the laceration of the serous and muscular tunics of the large and small bowels was induced, it is impossible to conjecture; nor is it easy to determine how far, or in what degree, it influenced the fatal event.

In two experiments of this kind by Dr. Smith of St. Croix, the results were of the most gratifying nature. In one, the excised portion of the small intestine—probably the ileum—measured two inches; in the other, two inches and a half. In both cases he made use of four interrupted sutures, placed at equal intervals, with the ends cut off at the knots. The animals were killed on the twentieth day, when the union was found to be so perfect that it was difficult to discover the seat of the injury. In one, all the ligatures were detached; in the other, one still remained.

The results of these observations and experiments are in the highest degree interesting, as they tend to establish an important practical precept. Cases occasionally occur in which the bowel is so much injured, cut, bruised or lacerated, as to be inevitably followed by gangrene, if the parts be not promptly excised, and treated in conformity with the principles here laid down. In extensive mortification from strangulation it becomes, as we have already seen, a question whether the affected portion should be removed by the knife, or the separation of it be intrusted to the efforts of nature. In the latter case, even supposing that the patient would run no risk from the effusion of fæcal matter into the peritoneal sac, he would still be subjected to that most loathsome of all diseases, an artificial anus; in the former, the injured structures would be placed in the same relations as those of a common incised wound, and the chances of recovery would therefore be incomparably greater. In intus-susception, where one portion of bowel falls into another, and where the included piece is finally detached by sloughing, nature performs the same operation precisely that the surgeon does under the circumstances in question, with the difference merely that she is much longer in accomplishing her object; which, however, is not less effectual in the end. The practice, then, would seem to be sanctioned, not only by reason and analogy, but by experiments on the inferior animals and observations on the human subject.

Would it be good practice, in extensive longitudinal or obliuqe wounds, to excise the affected part, and treat the case like one in which the tube is completely divided in the first instance? My opinion is that it would, especially where the opening is more than two inches in length. My reason for this conclusion is, that wounds of this extent require an unusually long time to heal, that the canal may become permanently contracted, and that the adhesive process is rarely so perfect as when the aperture is smaller. In addition to this, as was before remarked, there must necessarily be more irritation from the great number of sutures, to say nothing of the immediately bad effects occasioned by the protracted manipulation necessary to apply them. In an experiment, the particulars of which are detailed in another page, and in which the wound was three inches and a half long, death was evidently produced by the ulcerative action of the adventitious substance which formed the bottom of the opening, and which was consequently in direct contact with the contents of the tube. The abnormal aperture was nearly the size of half a dime. The animal lived till the end of the thirteenth day, and was considered entirely out of danger, when the perforation occurred which led to his death. Altogether eleven sutures had been used, of which only two remained. This case, although a solitary one, is sufficient, I think, to show the impropriety of employing so many sutures, or, rather, the inexpediency of attempting to save the affected part in extensive injuries of the intestinal canal.

Littre, an old French surgeon, was of opinion that the best practice, when the bowel is completely severed, whether by accident or mortification, is to bring the superior end out at the external opening, for the purpose of establishing an artificial anus, and to return the other into the peritoneal cavity, having previously tied it to effect its obliteration. The inevitable result of such a procedure would be to consign the patient to a miserable existence, as it would deprive him of all chance of recovery, and leave him with an infirmity that renders him disgusting to himself and to those around. It really becomes a question, as has been justly observed by Mr. Lawrence, whether life itself be desirable, if burthened with the discharge of fæces through the groin or some other region.

A more rational and less objectionable method was proposed by La Peyronie. It consists in passing a double thread behind the wound through a fold of the mesentery, and retaining the ends of the bowel at the outer aperture, by fastening the extremities of the ligature to the surface of the abdomen with adhesive strips. This operation, like that of Littre, is always followed by an artificial anus; but, instead of being rendered incurable, as necessarily happens in the latter case, it generally yields to judicious management. Several examples in which this expedient was successfully resorted to are on record. I select the following as one of the most recent and interesting.

A man at the assault of Cairo, in 1799, was wounded by a ball in the abdomen, which entered on the right side, and perforated the ileum. The two ends of the bowel were ruptured, separated from each other, and tumefied; the superior being turned upon itself, so that it looked like the præpuce in paraphymosis, and caused complete obstruction of the tube. By four small incisions with the crooked scissors, Baron Larrey, the reporter of the case, divided the neck of the strangulated intestine, and restored it to its proper situation. He then passed a ligature into the portion of the mesentery corresponding with the two ends of the canal, which he returned as far as the edge of the wound, which he had previously taken care to dilate. After dressing the parts, he waited the result. For the first few days the symptoms were unpromising, but they gradually abated in severity, the alvine evacuations daily improved, and in about two months the ends of the ileum were in apposition and ready to adhere. The wound was afterwards dressed with a plug, according to the ingenious plan suggested by Desault, and the soldier ultimately left the hospital completely cured.*

In a case mentioned by La Peyronie himself, the patient was about sixty-three years of age, and the bowel was affected with mortification from strangulation. The whole of the sphacelated part was cut away, and a thread passed through the mesentery, by which the ends of the gut were kept in apposition with the external opening. The fæces were voided through the artificial anus until the thirty-sixth day, when they began to resume their natural route, and in four months the ulcer was completely healed. Subsequently, however, an abcess formed at the seat of the cicatrice, followed by a new rupture.†

---

* Memoirs of Military Surgery, translated by Dr. Hall, vol. i, p. 320.
† Boyer, Traité des Maladies Chirurgicales, T. viii, p. 136.

The practice commonly pursued by surgeons, when the bowel is mortified in its entire cylinder, is to pull it gently down, and make a large incision into it, to afford a free outlet to the fæces. The artificial anus thus established gradually diminishes in size, and after some months disappears, the alvine matter, in the meanwhile, resuming its natural route. Upon the propriety or impropriety of this practice it is not necessary here to insist. Further observation can alone settle the question. When there is much inflammation beyond the sphacelated parts, it would probably be wrong to pursue any other treatment; if, on the other hand, the tube is nearly, or quite sound, I should not hesitate to excise the mortified structures, and to approximate the ends by the suture, in the manner already explained.

## Figures and Tables

**Figure f1:**
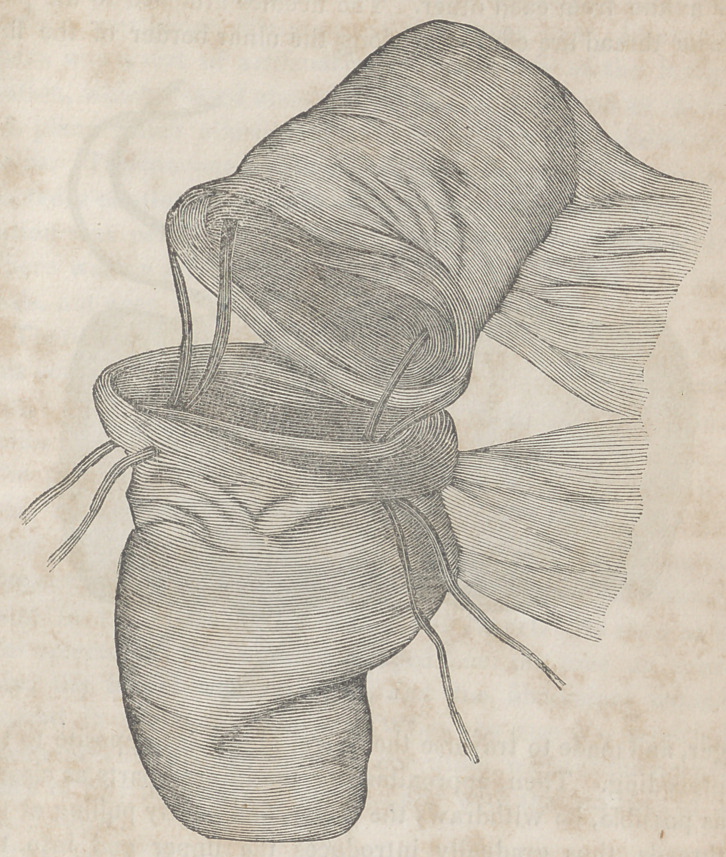


**Figure f2:**
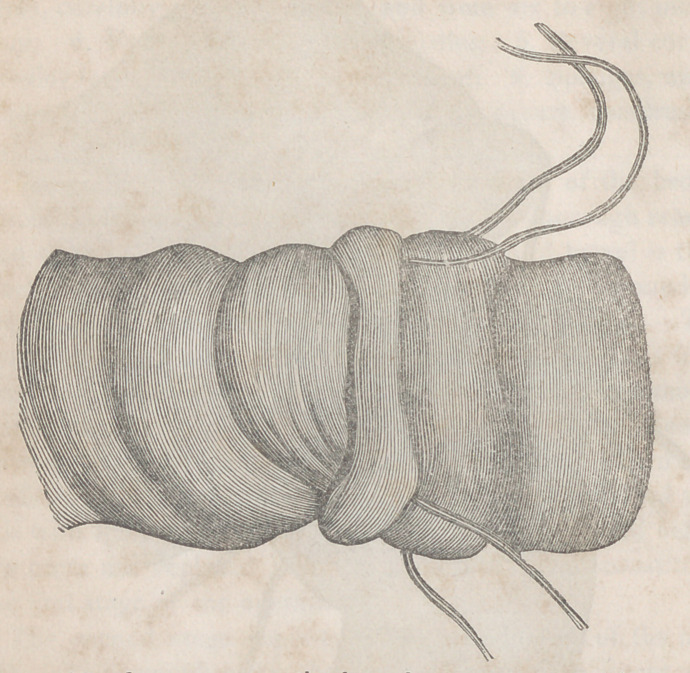


**Figure f3:**
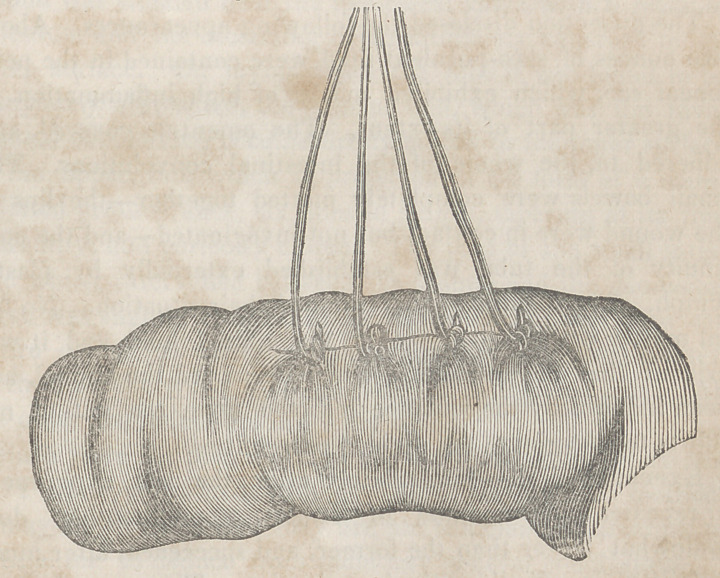


**Figure f4:**
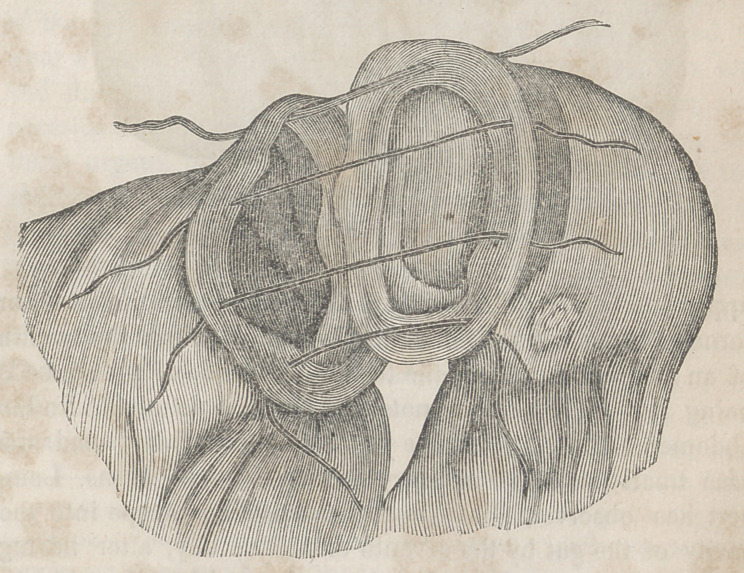


**Figure f5:**
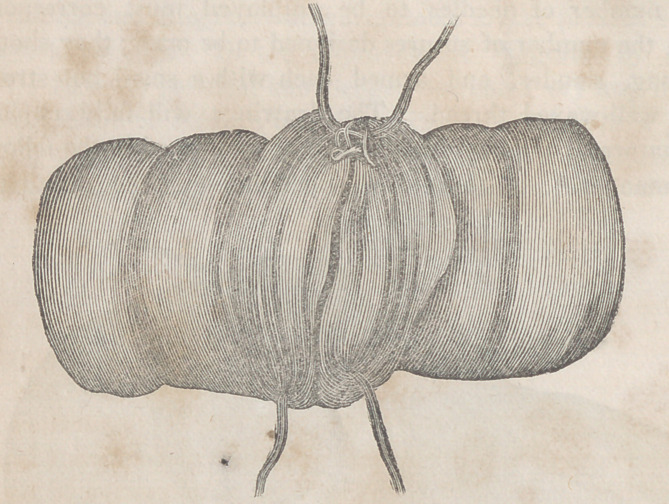


**Figure f6:**
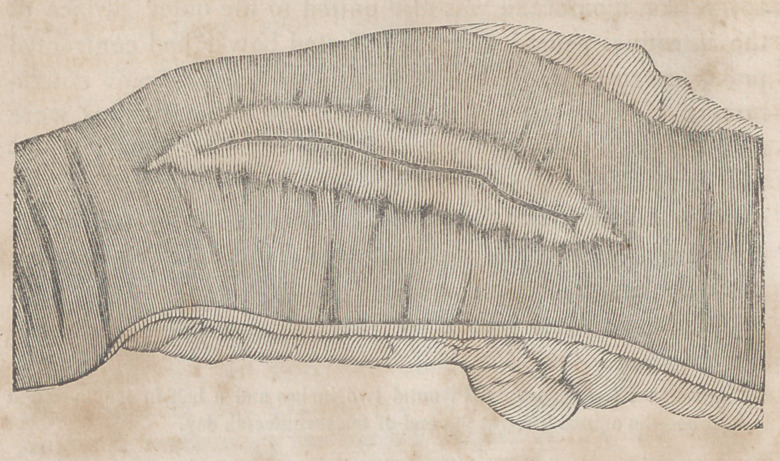


**1 f7:**
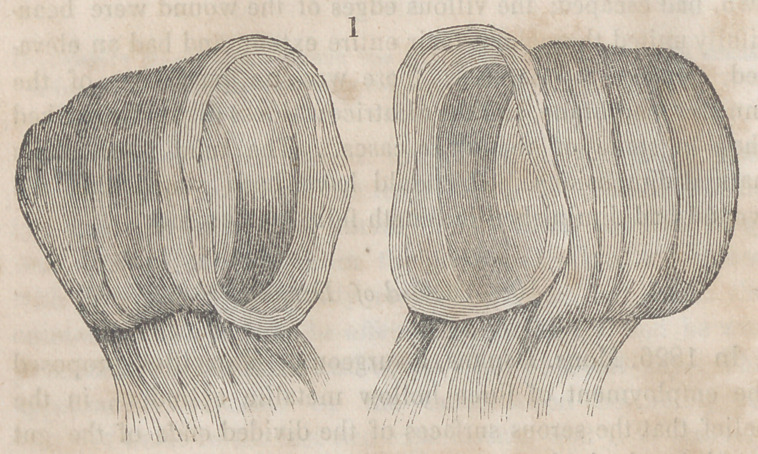


**2 f8:**
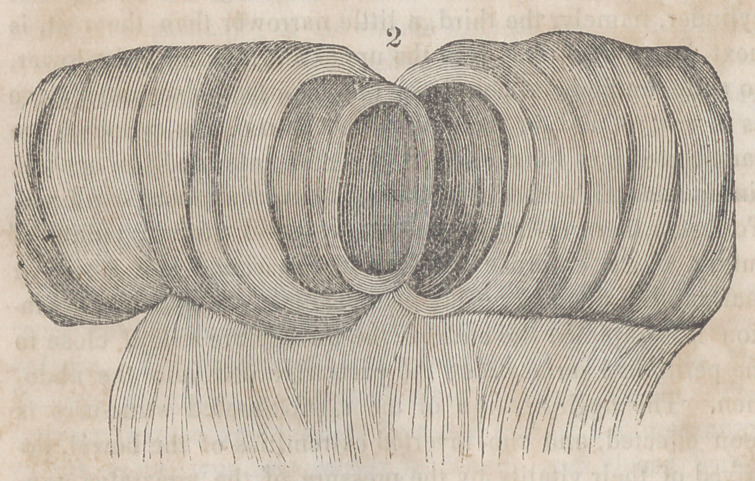


**3 f9:**
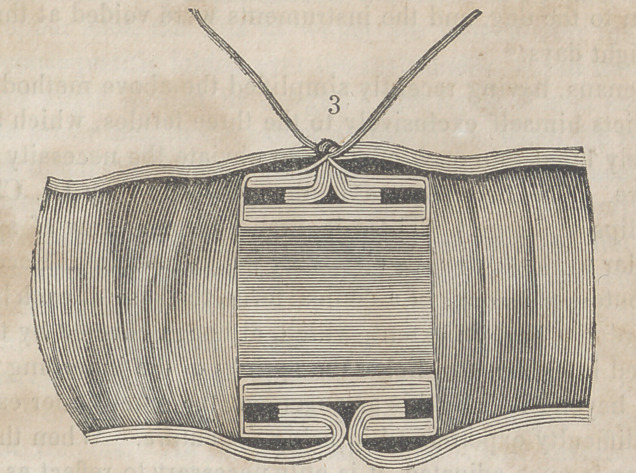


**4 f10:**
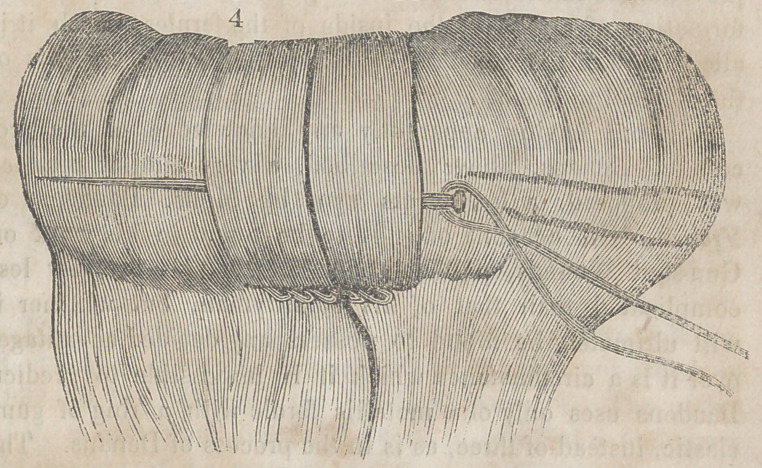


**1 f11:**
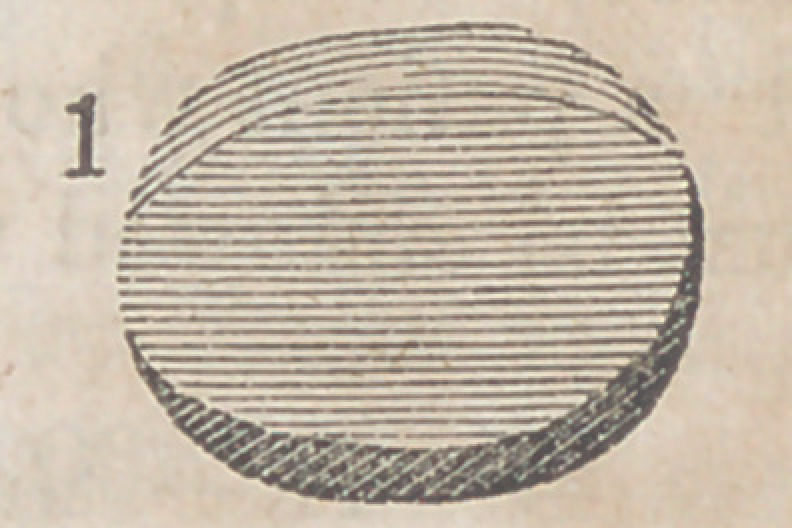


**2 f12:**
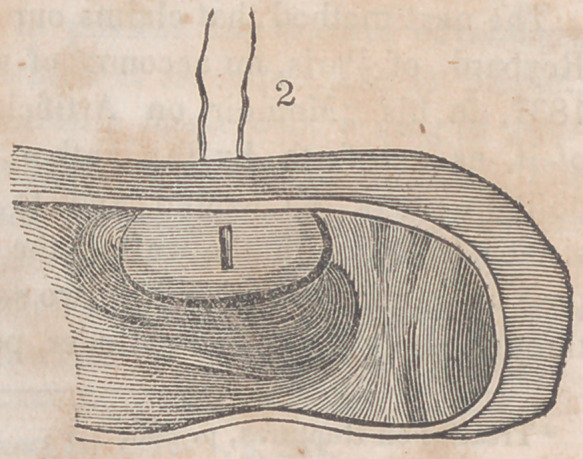


**3 f13:**